# Exploring Highly Conserved Regions of SARS-CoV-2 Spike S2 Subunit as Targets for Fusion Inhibition Using Chimeric Proteins

**DOI:** 10.3390/ijms232415511

**Published:** 2022-12-07

**Authors:** Daniel Polo-Megías, Mario Cano-Muñoz, Alberto G. Berruezo, Géraldine Laumond, Christiane Moog, Francisco Conejero-Lara

**Affiliations:** 1 Departamento de Química Física, Instituto de Biotecnología y Unidad de Excelencia de Química Aplicada a Biomedicina y Medioambiente (UEQ), Facultad de Ciencias, Universidad de Granada, 18071 Granada, Spain; 2 Laboratoire d’ImmunoRhumatologie Moléculaire, Institut National de la Santé et de la Recherche Médicale (INSERM) UMR_S 1109, Institut Thématique Interdisciplinaire (ITI) de Médecine de Précision de Strasbourg, Transplantex NG, Faculté de Médecine, Fédération Hospitalo-Universitaire OMICARE, Fédération de Médecine Translationnelle de Strasbourg (FMTS), Université de Strasbourg, F-67000 Strasbourg, France; 3 Vaccine Research Institute (VRI), F-94000 Créteil, France

**Keywords:** coronavirus, antivirals, inhibitors, COVID-19, calorimetry, protein engineering, protein design

## Abstract

Since the beginning of the COVID-19 pandemic, considerable efforts have been made to develop protective vaccines against SARS-CoV-2 infection. However, immunity tends to decline within a few months, and new virus variants are emerging with increased transmissibility and capacity to evade natural or vaccine-acquired immunity. Therefore, new robust strategies are needed to combat SARS-CoV-2 infection. The viral spike composed of S1 and S2 subunits mediates viral attachment and membrane fusion to infect the host cell. In this process, interaction between the highly conserved heptad repeat 1 and 2 regions (HR1 and HR2) of S2 is crucial and for this reason; these regions are promising targets to fight SARS-CoV-2. Here, we describe the design and characterization of chimeric proteins that structurally imitate the S2 HR1 region in a trimeric coiled-coil conformation. We biophysically characterized the proteins and determined their capacity to bind the HR2 region, as well as their inhibitory activity of SARS-CoV-2 infection in vitro. HR1 mimetic proteins showed conformational heterogeneity and a propensity to form oligomers. Moreover, their structure is composed of subdomains with varied stability. Interestingly, the full HR1 proteins showed high affinity for HR2-derived peptides and SARS-CoV-2 inhibitory activity, whereas smaller proteins mimicking HR1 subdomains had a decreased affinity for their complementary HR2 region and did not inhibit the virus. The results provide insight into effective strategies to create mimetic proteins with broad inhibitory activity and therapeutic potential against SARS-CoV-2.

## 1. Introduction

COVID-19 is the worst pandemic of this century and continues to be out of control, with recurrent waves of new infections worldwide. Soon after the identification of SARS-CoV-2 as the causative agent of the disease, hundreds of vaccines targeting the spike protein of the viral envelope were developed, and approximately 24 of them have been authorized for use [1]. Although the vaccines have been shown to be effective in protecting against infection and, in particular, in reducing the severity of the course of the disease in infected patients [2], immunity seems to decline over time within a few months [3], requiring repetitive administrations. Moreover, there is a continuous emergence of new virus variants capable of evading immunity acquired [4,5] either by infection or by vaccination [6]. In contrast to the vaccines, the therapeutic options for infected people are more limited, and the development of effective antiviral drugs has been slower and less effective. Nevertheless, some drugs are being tested, and a few have been approved for treatment of severe cases [7,8], including small-molecule compounds and monoclonal antibodies [9,10]. Therefore, the development of new therapeutic strategies is still urgently needed to fight the COVID-19 pandemic.

**Figure 1:**
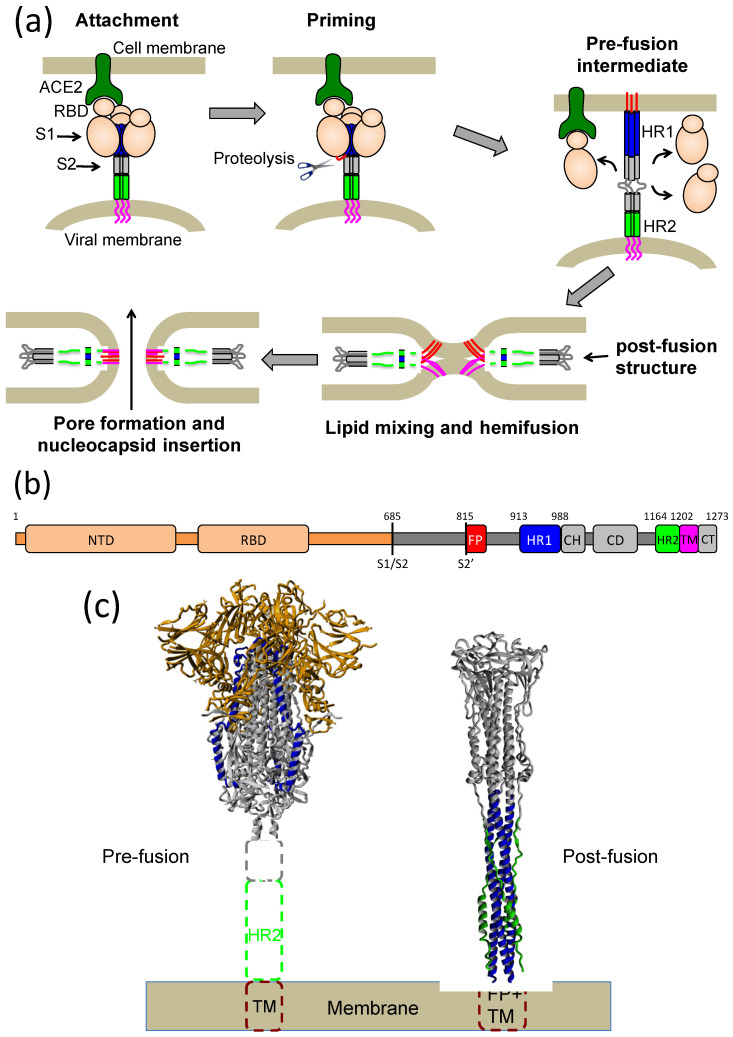
Mechanism of spike-mediated SARS-CoV-2 fusion. (**a**) Schematic representation of the putative steps of membrane fusion mediated by the spike protein. The HR1 and HR2 regions are indicated in blue and red, respectively, with the fusion peptide shown in red and the transmembrane region in magenta. (**b**) Primary structure of the full-length spike showing the sequence location of the various domains. S1/S2 and S2′ indicate the sites of proteolytic activation. (**c**) Structures of the prefusion spike in closed form and the S2 protein in post-fusion conformation. The models were built using the PDB entry 6VXX for the prefusion spike and 6M3W and 6LXT for the post-fusion structure. The dashed boxes indicate unresolved regions. The S1 subunits are indicated in orange in the prefusion structure. The HR1 and HR2 regions are indicated in blue and green, respectively, in each structure.

A highly desired therapeutic approach is to inhibit entry of the virus into cells. The entry mechanism of SARS-CoV-2 is, in general, similar to that of other enveloped viruses with class I fusion proteins, such as HIV, influenza virus or Ebola virus. SARS-CoV-2 must fuse its membrane with the cell membrane to introduce its genetic material and begin replication. The spike (S) protein that decorates the virus surface drives virus attachment to the cell and membrane fusion (Figure 1). The S protein is synthesized as a trimer that becomes cleaved into two subunits, S1 and S2, by proteolytic processing [11,12]. S1 is highly glycosylated and consists of the N-terminal domain (NTD), the receptor-binding domain (RBD) and two C-terminal domains (CTD). S2 contains a fusion peptide (FP), a fusion-peptide proximal region (FPPR), heptapeptide repeat region 1 (HR1), a central helix (CH), a connector domain (CD), heptapeptide repeat region 2 (HR2), a transmembrane domain (TM) and a short internal C-terminal tail (CT).

Attachment of the virus to the host cell occurs through binding of RBD to the angiotensin-converting enzyme-2 receptor (ACE2) [13]. Then, spike priming encompasses cleavage by host cell proteases at the S1/S2 and the S2′ sites [14]. The three S1 subunits cover an internal S2 trimer and lock it in a spring-loaded metastable prefusion conformation. After proteolytic priming and S1 shedding, the S2 trimer extends its central CH coiled coil towards its N terminus incorporating the HR1 region, projecting the FP outwards and inserting it into the membrane, exposing a hydrophobic groove between each pair of HR1 helices. Then, S2 folds onto itself, and each HR2 region binds onto each one of the HR1 grooves to form an energetically favorable six-helical bundle structure that facilitates viral and cell membrane apposition and fusion [15].

Owing to their key role in promoting membrane fusion, HR1 and HR2 are highly conserved and therefore constitute promising targets for the development of coronavirus treatments. The fusion process can be interfered with by peptides derived from HR2 [16,17,18], as observed for other coronaviruses [19] and for gp41-mediated HIV-1 fusion [20]. HR1-derived peptides are less potent inhibitors targeting HR2 [16,19] but show strongly increased inhibitory activity if they have a stabilized trimeric coiled-coil structure exposing a stable HR1 groove [21,22,23], similar to HIV-1 fusion inhibitors based on the HR1 region of gp41, the HIV protein equivalent to S2 [24,25,26].

Coiled coils are extremely versatile structures formed by polypeptides with seven-amino acid repeats, also known as heptad repeats (HR), in which the first (a) and fourth (d) of the seven positions are typically occupied by a hydrophobic amino acid (most frequently Val, Leu or Ile) [27]. In these supercoiled α-helical structures, each helix interacts with the neighboring structures via a so-called ‘knobs-into-holes’ interaction [28], whereby an amino acid side chain of a helix (a knob) packs into a cavity (a hole) created by four side chains of a facing helix. In addition, charged amino acids, such as lysine (K) and glutamic acid (E), are frequently placed at (e) and (g) positions to encourage inter-helix salt bridges flanking the hydrophobic core. HR sequences can adopt parallel or antiparallel dimeric, trimeric and tetrameric arrangements depending on the particular amino acids occupying each core position [29].

We previously made use of this structural versatility of coiled coils to design chimeric proteins that accurately mimic the structure of the central coiled-coil HR1 trimer of HIV gp41 [24]. In these designed proteins, one of the HR1-parallel helices was upturned and its sequence was reversed to create an antiparallel trimeric bundle so that the three helices could be linked by two short loops in a single polypeptide chain with a helix–loop-helix–loop–helix topology. The helical bundle was then stabilized by a series of engineered amino acid changes, creating favorable electrostatic interactions. These chimeric proteins folded spontaneously and stably as expected, accurately imitated an exposed HR1 groove between the two parallel HR1 helices and tightly bound to gp41 HR2 peptides [30,31]. Moreover, these mimetic proteins showed potent and broad inhibitory activity against a variety of HIV-1 strains [24,30,31,32,33].

Given the structural and mechanistic similarities between HIV and SARS-CoV-2 fusion proteins, we hypothesized that similar chimeric single-chain coiled coils could be designed to imitate the HR1 region of S2 and that these mimetic proteins could interact with the highly preserved HR2 regions and thereby inhibit SARS-CoV-2 infection. Here, we describe the design and engineering of several chimeric proteins and their detailed biophysical characterization. We describe the molecular properties of these chimeric proteins and their capacity to acquire their expected folded structure, their stability against denaturation and their binding affinity for the HR2 region. We also investigated their activity against SARS-CoV-2 cell infection in vitro. The results serve to guide the design of new chimeric proteins that may serve as antivirals and are also of general interest in protein design and engineering of coiled-coil proteins.

## 2. Results

### 2.1. Design of an Antiparallel Trimeric Coiled-Coil Protein Mimicking the S2 HR1 Trimer

**Figure 2:**
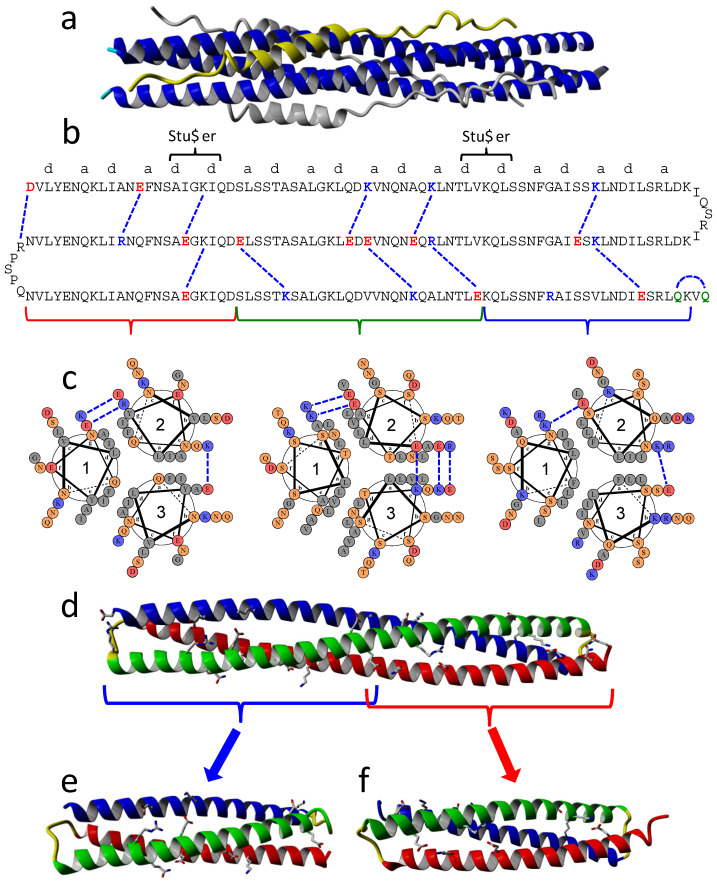
Design of CoVS-HR1 proteins. (**a**) Structure of the core post-fusion complex between HR1 and HR2 (PDB id. 6LXT [18]) used as a design template. The three central HR1 helices are represented in blue, and the three external HR2 regions are indicated in grey and yellow. (**b**) Sequence and topology of the L1 variant indicating the engineered amino acid substitutions (highlighted with colors) and the stabilizing interactions (with blue dashed lines). The ‘a’ and ‘d’ characters on top of the first sequence stretch indicate the corresponding positions in the HR1 heptad repeat. Two stutters in the canonical repeats are indicated. (**c**) Helix-wheel diagrams corresponding to sections of the coiled coils are indicated with brackets under (**b**). The diagrams were generated with Drawcoil (https://grigoryanlab.org/drawcoil/, accessed on 2 June 2022). The blue dashed lines indicate the engineered stabilizing interactions in each molecule. (**d**) Ribbon representation of the model of the full-length HR1-mimetic proteins (CoVS-HR1-L1 and CoVS-HR1-L2). The parallel HR1 helices 1 and 3 are indicated in blue and red, respectively, and the reversed helix 2 is indicated in green. The loops connecting the helices are colored yellow. The side chains of amino acids engineered to stabilize the antiparallel fold are represented by sticks. (**e**,**f**) Ribbon models of the shortened CoVS-HR1-N (**e**) and CoVS-HR1-C miniproteins (**f**).

Following a similar design strategy as previously used for HIV-1 gp41 HR1 mimics [24], we used as template the previously published structure of the HR1-HR2 complex of S2 (Figure 2a) (PDB id. 6LXT) [18]. We took the three central HR1 helices and homology-modeled an antiparallel helix by reversing its sequence and spatial orientation relative to the original HR1 helix. The reversed helix was aligned to one of the original helices, superposing the side-chain β carbons of the core (a) and (d) residues in order to minimize the perturbation of the knobs-into-holes core packing in the coil. The reversed helix replaced the original parallel helix. Then, side-chain clashes were corrected by modifying the side-chain rotamers, and the energy of the model was minimized. With this arrangement, the C-terminal and N-terminal ends of the reversed helix were close enough in space to the opposite ends of the original parallel helices so that they could be linked using short polypeptide segments, thus creating a single polypeptide chain.

The loops were designed using the build loop tool of YASARA Structure software [34], which searches the PDB for residue segments of varying lengths that best fit between two selected anchors points. We built two versions of five-residue loops connecting each pair of helices, using Asn 914 and Lys 986 HR1 residues as anchor points. The amino acid sequences of the loops were rationally optimized to avoid side-chain clashes, improve local interactions and reduce the exposed hydrophobic surface. This process generated two variants code-named CoVS-HR1-L1 and CoVS-HR1-L2.

Then, several amino acid changes along the helices at the (e) and (g) positions of the heptad repeats were rationally engineered to create stabilizing electrostatic interactions or hydrogen bonds (Figure 2b,c). The changes were selected by visual inspection of the side chains in the model structure searching for appropriate side-chain orientations and distances to allow for the establishment of a favorable interaction. To preserve the HR2-binding capacity, the amino acids of the two parallel HR1 helices that form the HR2-binding groove where not modified. The final models were energy-minimized (Figure 2d). The L1 and L2 variants differ only in the loop sequences (Table S1).

The DNA encoding each protein sequence was synthesized and inserted into a suitable expression vector, including an N-terminal Met and a C-terminal 6×His-tag. The proteins were overexpressed in *E. coli* cells with high yields and purified to homogeneity by two-step chromatography (see Section 4 for details).

### 2.2. Biophysical Characterization of the CoVS-HR1 Proteins

**Figure 3:**
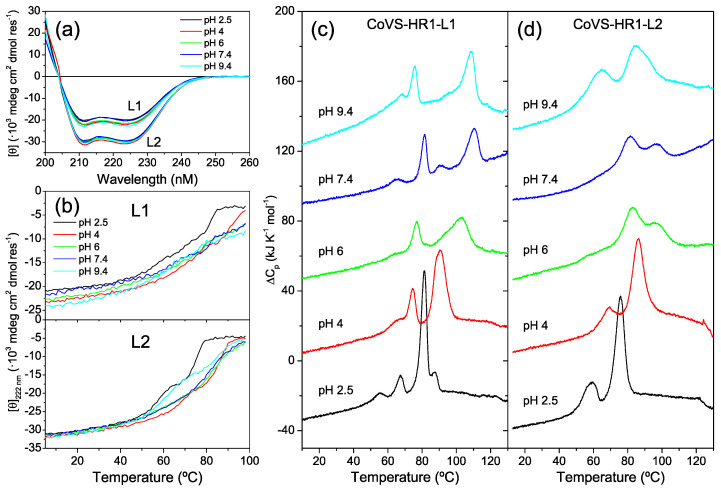
Structure and stability of the CoVS-HR1 L1 and L2 proteins. (**a**) Far-UV CD spectra of L1 and L2 measured with 15 μM protein solutions at varied pH and 25 °C. The spectra were normalized as mean residue molar ellipticity. (**b**) Thermal scans monitoring the CD ellipticity at 222 nm under the same conditions as in panel (**a**). (**c**,**d**) DSC thermograms of L1 (**c**) and L2 (**d**) proteins measured at 30 μM at varying pH values, as indicated near each curve. The thermograms are artificially displaced along the vertical axis for the sake of clarity.

The L1 and L2 proteins were highly soluble in standard buffers. The far-UV circular dichroism (CD) spectra indicated an α-helical structure (Figure 3a), in agreement with the models. The apparent α-helix percentage estimated based on the negative ellipticity signal at 222 nm [35] was considerably higher for L2 (80–83%) than for L1 (54–62%). Thermal unfolding experiments carried out by monitoring the CD signal at 222 nm (Figure 3b) showed complex unfolding curves. More detailed thermal unfolding experiments were performed by differential scanning calorimetry (DSC) (Figure 3c,d), showing multiple transitions in the unfolding processes for both proteins. Whereas L1 showed as many as four partially overlapping transitions, L2 displayed a simpler unfolding, with only two major peaks, but a faint and broad shoulder was also visible at lower temperature at intermediate pH. The thermal transitions observed by DSC imply sequential loss of the α-helix structure, as observed in the CD thermal scans (Figure 3b). The highest thermal stability was observed at pH 7.4 for both proteins.

The reversibility of the unfolding transitions at pH 7.4 was checked by performing consecutive DSC scans up to temperatures just past each transition (Figure S1a,b). The unfolding profile of the L1 protein showed a complex behavior, and all the peaks displayed only partial reversibility. Moreover, the unfolding processes in all the peaks were kinetically controlled according to a clear dependence on the scan rate used in the DSC experiments (Figure S1c). In contrast, the broad shoulder and the main unfolding peak of L2 were independent of the scan rate (Figure S1d) and reproduced in consecutive scans, indicating equilibrium unfolding processes. However, the high-temperature transition of L2 indicates kinetic control, and after this transition, the protein was irreversibly denatured.

**Figure 4:**
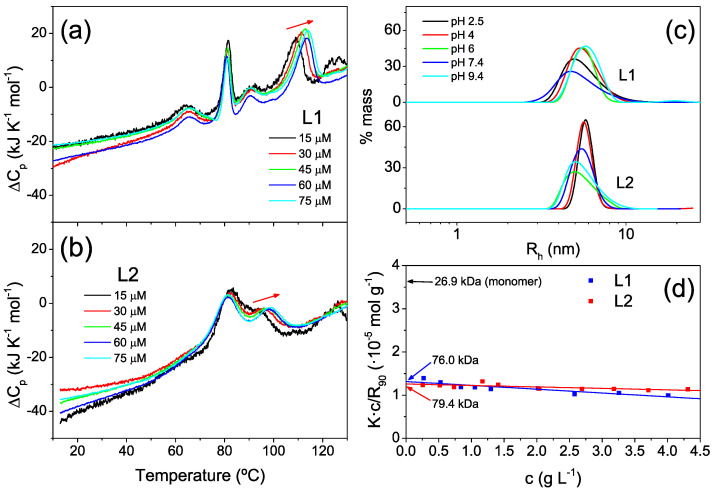
Effect of protein concentration on stability and molecular size characterization. (**a**,**b**) DSC thermograms measured for L1 (**a**) and L2 (**b**) proteins at pH 7.4 and varying protein concentrations between 15 and 75 μM. The red arrows highlight temperature shifts of the unfolding transitions associated with increased concentration, suggesting oligomer dissociation accompanying unfolding. (**c**) Distributions of hydrodynamic radii measured by DLS for L1 and L2 proteins at varying pH values and 25 °C. (**d**) Debye plots obtained by scattering intensity measurements with protein samples of varying concentrations at pH 7.4 and 25 °C according to Equation (1) (see Section 4). The intercepts of the linear regressions correspond to the inverse of the weight-averaged molecular weights (1/*M_w_*) of the particles in solution. The theoretical intercept (1/*M_w_*) for the protein monomers is also indicated.

To further understand these intricate unfolding profiles, we carried out DSC experiments at varied protein concentrations, ranging from 15 μM to 75 μM (Figure 4a,b). Whereas the peaks at lower temperatures did not change with the protein concentration, the last peaks shifted towards higher temperatures with increased concentration, which is indicative of a dissociation process accompanying unfolding [36]. These results suggest the presence of highly stable oligomeric species in the protein samples. The invariability of the transitions at lower temperatures with concentration indicates partial unfolding processes not involving changes in the oligomerization state.

To confirm the presence of oligomers, we carried out dynamic light scattering (DLS) measurements. The hydrodynamic radii measured at varying pH values (Figure 4c) ranged between 4.8 and 5.7 nm—significantly higher than expected for monomeric proteins (around 3.3 nm), as estimated with Hydropro software [37] using the design models. The presence of oligomers was confirmed by measurements of the static scattering intensity of samples at pH 7.4 with varying protein concentrations (Figure 4d). The measured average molecular weights (*M_w_* = 76.0 and 79.4 kDa) were compatible with trimeric species, compared to the theoretical molar masses of the protein monomers of 26.9 and 26.8 kDa, respectively, in accordance with the concentration effects observed in the high-temperature DSC transitions.

Overall, these results allowed us to conclude that the CoVS-HR1 mimetic proteins do not have a fully cooperative structure but contain subdomains of varying stability. Moreover, the proteins tend to self-associate as trimers. L2 appeared to be more structured than L1 and showed fewer transitions, highlighting the importance of the loop composition in the folding of these proteins.

### 2.3. Binding of the CoVS-HR1 Proteins to HR2-Derived Peptides

**Figure 5:**
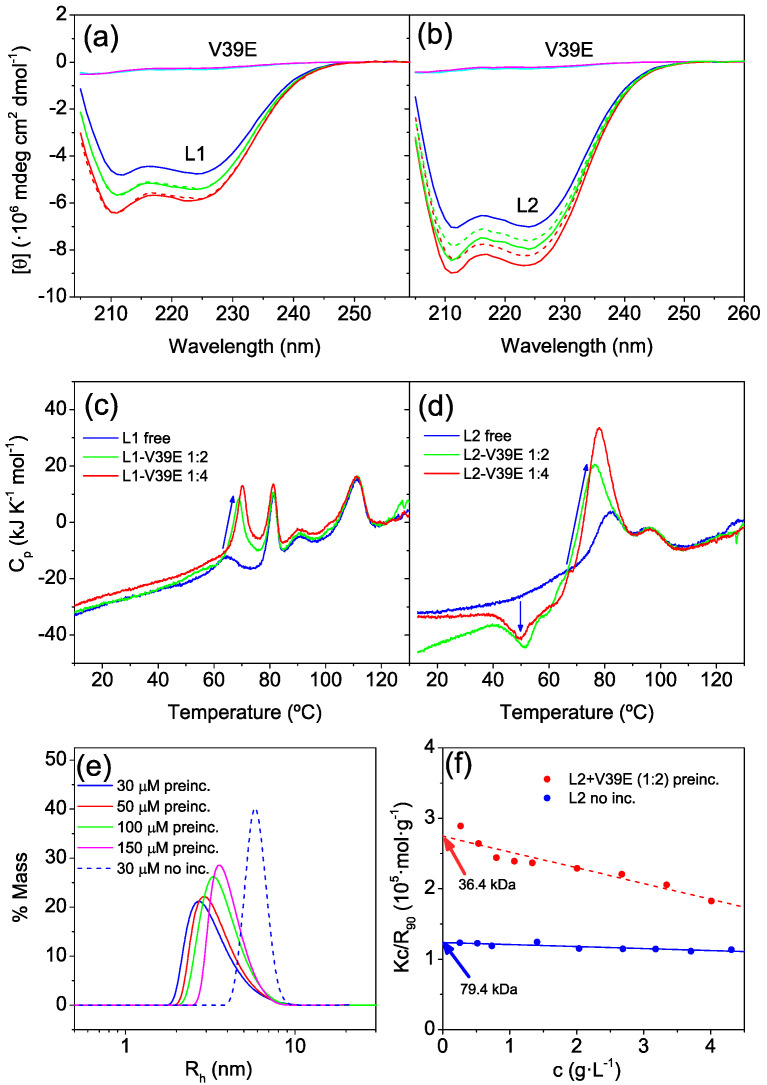
Effect of binding of the HR2-derived peptide V39E on the structure and stability of the L1 and L2 proteins. (**a**,**b**): Far-UV CD spectra of free L1 (**a**) and L2 (**b**) proteins (blue) and their mixtures with V39E peptide in 1:2 (green) and 1:4 (red) molar ratios. Spectra were recorded at pH 7.4 and 25 °C. The CD data were normalized per mole of protein monomer. The spectra of free V39E peptide measured at 30 μM and 60 μM under the same conditions are shown as cyan and magenta lines, respectively. The theoretical CD spectra calculated by the summation of spectra of the free proteins and the added free peptide are also indicated as dashed lines for comparison. (**c**,**d**): DSC scans measured at pH 7.4 for the free L1 (**c**) and L2 (**d**) proteins at 30 μM and in presence of V39E peptide at 1:2 and 1:4 molar ratios. The thermograms measured with the free V39E peptide under identical conditions were subtracted to remove its contribution to the heat capacity and observe the net effects of binding on the protein heat capacity. The blue arrows highlight the changes in the transitions produced by peptide binding. (**e**) Hydrodynamic radius distributions measured by DLS with L2:V39E mixtures at a molar ratio of 1:2 at varying protein concentrations as indicated. The mixtures were preincubated at 55 °C for 15 min before measurement at 25 °C. The equivalent measurement with a non-preincubated mixture is also shown with dashed line. (**f**) Debye plot corresponding to SLS measurements at 25 °C with L2:V39E mixtures of varying concentrations preincubated at 55 °C for 15 min (red). The Debye plot corresponding to free L2 is shown in blue for comparison.

To explore whether these proteins can bind to their HR2 target, we measured the far-UV CD spectra of mixtures between the proteins and a synthetic peptide encompassing residues Val1164-Glu1202 (named V39E) that corresponds to the HR2 region of the S protein (See Table S1), taking advantage of the fact that the HR2 peptide is mainly disordered in isolation but acquires a partial helical structure upon binding to HR1 [15] (Figure 5a,b). Whereas the increase in helical structure was minimal in L1-V39E mixtures, the L2-V39E mixtures showed larger increases in α-helix structure, suggesting stronger binding. According to DLS measurements at 25 °C, the presence of the V39E peptide in the mixtures produced only some broadening of the size distributions of the particles (Figure S2).

DSC scans with protein–peptide mixtures were also carried out to explore whether a possible interaction of the V39E peptide with the proteins could alter any of the unfolding transitions (Figure 5c,d). The presence of the peptide in molar excess affected only the first unfolding peak among the four peaks observed in L1, with a temperature shift and a strong increase in its area. The stronger endothermic peak induced by the increase in peptide concentration denoted the unfolding and dissociation of the protein–peptide complex. It appears that peptide binding specifically stabilized the less stable subdomain, whereas the other peaks did not become affected by the peptide. In the case of L2, the presence of the V39E peptide produced a more complex effect, with the appearance of a clear exothermic peak around 52 °C and a strong endotherm around 75 °C. The exothermic effect indicated a non-equilibrium time-dependent process, as heating cannot induce equilibrium exothermic processes [38], suggesting a slow transition associated protein–peptide complex formation from a metastable state. The intense endothermic peak at approximately 75 °C could also be associated with peptide dissociation and unfolding of the less stable L2 subdomain. The larger area of this transition suggests a stronger peptide binding for L2 compared to L1.

To further investigate the potential influence of the exothermic effect induced by peptide binding on the oligomerization state of the L2 protein, we preincubated L2-V39E mixtures at varying protein concentrations and a 1:2 protein:peptide molar ratio for 15 min at 55 °C, just after the exothermic peak. Then, the mixture was cooled to 25 °C, and the hydrodynamic radius was measured by DLS (Figure 5e). We observed considerable decreases in the apparent R_h_, which ranged between 2.7 nm and 3.6 nm depending on the protein concentration, compared to the R_h_ of 5.8 nm in a non-preincubated mixture. Moreover, a Debye plot corresponding to the scattering intensities measured with the preincubated L2-V39E mixtures at varying concentrations showed a considerable reduction in the average *M_w_* compared to the free L2 protein (Figure 5f), clearly indicating that the V39E peptide binds to and stabilizes the L2 monomer, although this process is slow at room temperature, accelerating at temperatures above ≈40–50 °C. Like in L1, the peptide did not alter the high-temperature unfolding transition of L2. These results show that the two proteins can bind the HR2 peptide and that the interaction mainly involves the less stable subdomain of the proteins.

**Figure 6:**
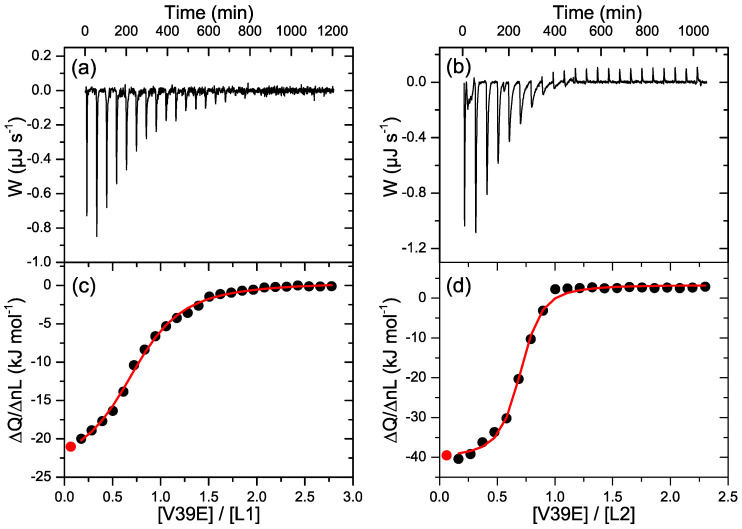
Isothermal titration calorimetry (ITC) experiments of the binding of the HR2 V39E peptide to the CoVS-HR1 proteins. (**a**,**b**): Baseline-corrected ITC thermograms measured for the titration of L1 (**a**) and L2 (**b**)m showing exothermic peaks associated with protein–peptide binding. (**c**,**d**) Binding isotherms for L1 (**c**) and L2 (**d**) calculated according to the respective thermograms. The plots show the binding heats normalized per mole of added peptide versus the peptide-to-protein molar ratio. The black symbols represent the experimental heats, and the red lines represent the fittings using a binding model of n identical and independent sites.

The binding of the V39E peptide to the two protein variants was also studied by ITC (Figure 6). The ITC thermogram measured at 25 °C for the titration of L1 with V39E showed sharp negative peaks, indicating an exothermic interaction (Figure 6a). The ITC thermogram measured for L2 with the same V39E peptide also showed exothermic binding, but the peaks were broader and recovered the baseline slowly as a result of slow heat release (Figure 6b), which is indicative of slow binding, as also observed in the DSC scans. The sigmoidal binding isotherms were analyzed using a model of n independent and identical sites, with apparent binding stoichiometries of less than 1 for the two proteins. The apparent binding affinities and binding enthalpies are presented in Table 1. The V39E binding affinity and enthalpy of L1 were lower than those of L2, in agreement with the above-described results. The binding parameters should not be considered accurate thermodynamic magnitudes characterizing the binding processes, as the above-described self-association processes of the proteins could significantly affect the ITC data. Nevertheless, these results show that L2 has a higher HR2-binding affinity than L1.

### 2.4. Design of Shortened CoVS-HR1 Proteins Mimicking HR1 Subdomains

To further understand the subdomain organization proposed for the CoVS-HR1 proteins and delimit the region of interaction of the HR2 peptide, we designed two shortened versions of the proteins, each encompassing about half of the HR1 region (Figure 2e,f). The design of these shortened proteins was derived from the L2 model. The three helices where clipped at positions near the center of the molecule, and the newly generated N-and C-chain ends where connected with loops. To create a miniprotein mimicking the N-terminal half of HR1, the L2 model was cut after residues Q41, N110 and Q197; then, residues Q41 and Q111 were linked using the same five-residue segment previously used to create the first L2 loop (GQLNP). This protein was called CoVS-HR1-N. A similar process was used to design a miniprotein encompassing the C-terminal half of HR1 (CoVS-HR1-C). In this case, the cut was made after residues A29, G119 and L188. The second and third helices were connected between G119 and G198 by the same five-residue loop used for the L2 variant (QILGP). The glycine anchor residues were substituted for asparagine to favor capping of the helix ends. Because the resulting CoVS-HR1-C protein does not contain an aromatic side chain, a tryptophan residue was added at the C terminus to confer UV absorption and facilitate concentration measurement. Finally, the two models were energy-minimized to validate their stability. The sequences of the proteins are presented in Table S1. Similar to their longer parent molecules, the two miniproteins containing an N-terminal Met and a C-terminal His-tag were cloned in expression vectors, produced by *E. coli* overexpression and purified with high yields.

### 2.5. Biophysical Characterization of the HR1 Short Miniproteins

**Figure 7:**
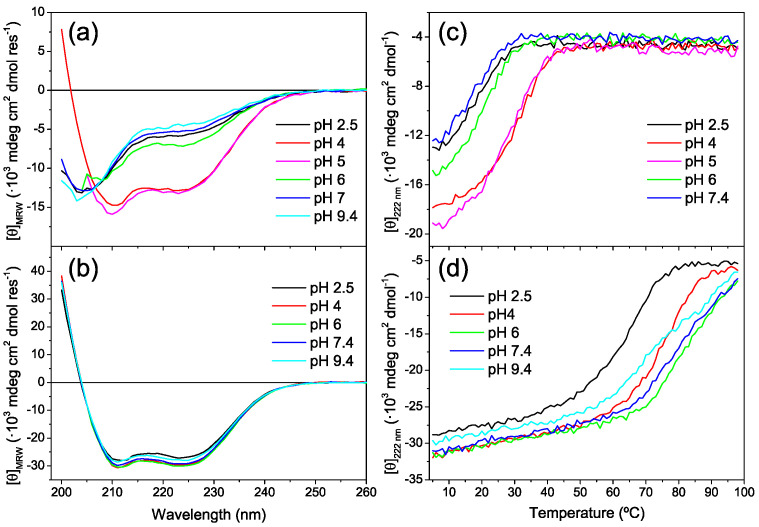
Secondary structure and stability of shortened CoVS-HR1 miniproteins. (**a**,**b**) Far-UV CD spectra of the CoVS-HR1-N (**a**) and CoVS-HR1-C (**b**) miniproteins measured at 25 °C at varying pH values. (**c**,**d**) Thermal scans monitored by CD at 222 nm at varying pH for the N (**c**) and C (**d**) miniproteins. Experiments were carried out with 15 μM samples.

At 25 °C, CoVS-HR1-N showed CD spectra typical of a partially disordered protein (Figure 7a). The protein displayed increased α-helicity (36%) only at mild acid pH, whereas at pH 2.5 and above pH 6, the protein was largely disordered (% α helix between 14% and 21%). The percentage of α-helix structure increased when the temperature was reduced to 5 °C, suggesting marginal stability for this protein. In contrast, CoVS-HR1-C showed a highly α-helical structure at 25 °C (approximately 84% between pH 4 and pH 7.4 and slightly decreasing at extreme pH values) (Figure 7b). Thermal unfolding monitored by CD at 222 nm confirmed that the N miniprotein is marginally stable, with apparent unfolding temperatures ranging between 17 °C and 29 °C, depending on the pH (Figure 7c), whereas the C miniprotein unfolded at higher temperatures, with maximum stability at pH 6 (Figure 7d).

**Figure 8:**
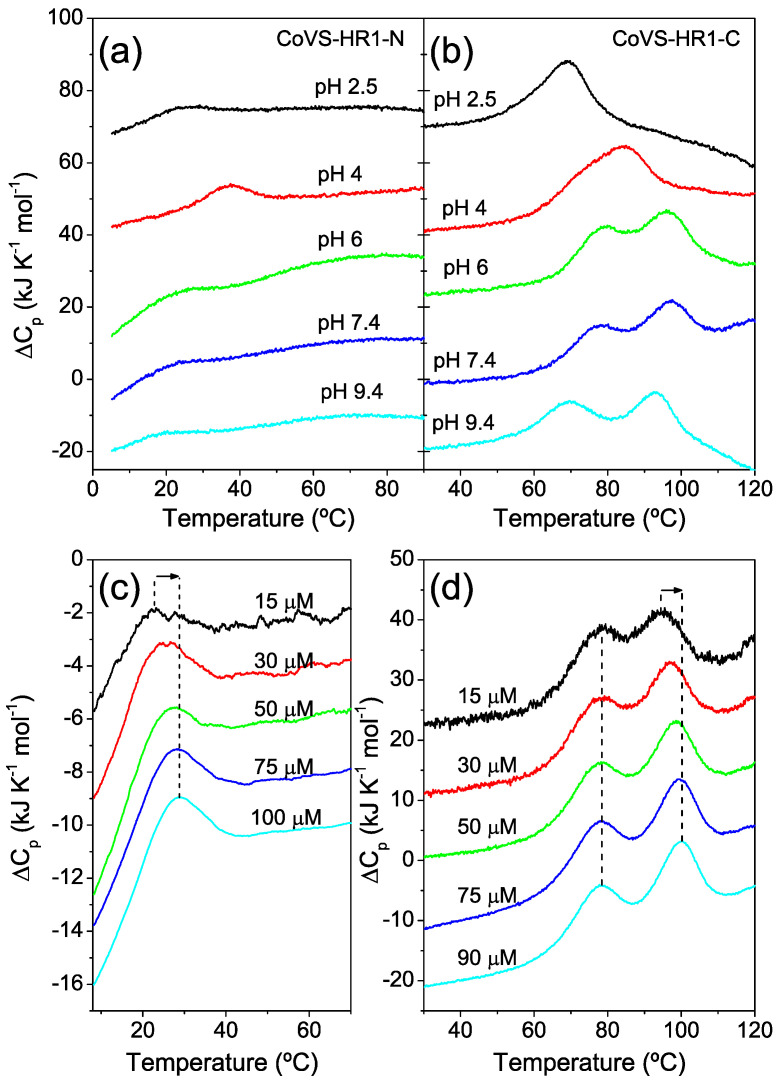
Thermal unfolding of shortened CoVS-HR1 miniproteins. (**a**,**b**) DSC scans of CoVS-HR1-N (**a**) and CoVS-HR1-C (**b**) measured at varying pH values as indicated next to each curve. The protein concentration was 30 μM in all experiments. (**c**,**d**) Effect of protein concentration on the DSC thermograms at pH 7.4. Al experiments were recorded at a scan sate of 90 °C h^−1^. The thermograms are artificially displaced along the vertical axis for the sake of clarity.

DSC scans with CoVS-HR1-N showed very weak unfolding peaks at low temperature, confirming the intrinsically low stability of the N miniprotein (Figure 8a). These faint thermal transitions are suggestive of a molten-globule-like state for this protein. The unfolding transitions measured at pH 5 and pH 7.4 increased their area and shifted to higher temperatures with increased protein concentration, suggesting self-association (Figure 8c and Figure S3a).

DLS measurements produced larger hydrodynamic radii than predicted for monomeric proteins (Figure S4a,b), indicating the presence of oligomeric species for both proteins. The Debye plot of CoVS-HR1-N obtained with scattering intensities measured at 5 °C at varying concentrations shows an *M_w_* of 24.8 kDa (Figure S4c) intermediate between the molar masses of the monomer (15.9 kDa) and the dimer (31.8 kDa). At pH 5 and 5 °C, the protein appeared to form mainly dimers (Figure S4c), as supported by the effect of protein concentration on the DSC unfolding curves at pH 5, which conforms to a two-state N_2_ ⇔ 2U model (Figure S3a). The N miniprotein is therefore intrinsically unstable, and its self-association as dimeric species appears to stabilize its folded helical structure.

On the other hand, the C miniprotein was much more stable and showed two partially overlapping unfolding transitions in the DSC scans (Figure 8b) reminiscent of those observed for the complete CoVS-HR1-L2 protein (see Figure 3d). Whereas the low-temperature peak was independent of the protein concentration, the high-temperature peak showed a marked dependency, indicating a coupling of unfolding with oligomer dissociation (Figure 8d). These DSC curves were globally fitted using a N_2_ ⇔ I_2_ ⇔ 2U unfolding model (Figure S3b), whereas a similar model assuming trimers did not adequately fit the curves. The Debye plot of the C miniprotein at pH 7.4 and 25 °C was curved at low concentration (Figure S4d). Extrapolation of the linear part indicated an *M_w_* value closely corresponding to a trimer, whereas at low concentrations, the plot tended towards intermediate values between the expected *M_w_* of the monomer and the dimer, suggesting the existence of a dynamic self-association equilibrium.

These results clearly indicate that the low-stability subdomain of the HR1 mimetic proteins corresponds to the N-terminal half, whereas the C-terminal half contains the high-stability subdomains. Moreover, the C domain seems to mediate self-association as highly stable oligomeric species.

### 2.6. Binding of HR2 to HR1 Subdomains

Binding of HR2-derived peptides to the N and C miniproteins was monitored by CD, DSC and ITC using synthetic peptides corresponding to the complementary HR2 regions of each HR1 half (see Table S1). For CoVS-HR1-N, we used a peptide encompassing HR2 residues Val1176-Glu1202 (named V27E), and for CoVS-HR1-C, we used a peptide with HR2 residues Val1164-Glu1182 (named V19E). CD spectra of the protein–peptide mixtures did not produce clear results, even for 1:8 protein peptide mixtures (Figure S5a,b). For instance, the mixtures of the N protein with the V27E peptide resulted in CD spectra with less negative ellipticity than predicted for the theoretical sum of spectra of the free molecules, possibly because the free V27E peptide already showed significant ellipticity owing to its partial helical structure. However, thermal scans monitored by CD at 222 nm showed clear stabilization of the CoVS-HR1-N miniprotein in the presence of increasing amounts of V27E (Figure S5c), suggesting binding. In contrast, the CoVS-HR1-C miniprotein was not stabilized significantly in the presence of variable amounts of the V19E peptide (Figure S5d).

DLS measurements of CoVS-HR1-N at 5 °C in the presence of the V27E peptide in a 1:8 molar ratio showed a marked reduction in the apparent hydrodynamic radius (Figure S6a), suggesting that the binding of the peptide specifically stabilizes the monomer. In the case of CoVS-HR1-C, the presence of the complementary V19E peptide in a 1:8 molar ratio also shifted the apparent hydrodynamic radius to smaller values (Figure S6b) but to a lesser extent than for the mixtures of CoVS-HR1-N and V27E. This slight shift may be due the result of an excess of small peptide molecules that contributed to the reduction in the average R_h_.

**Figure 9:**
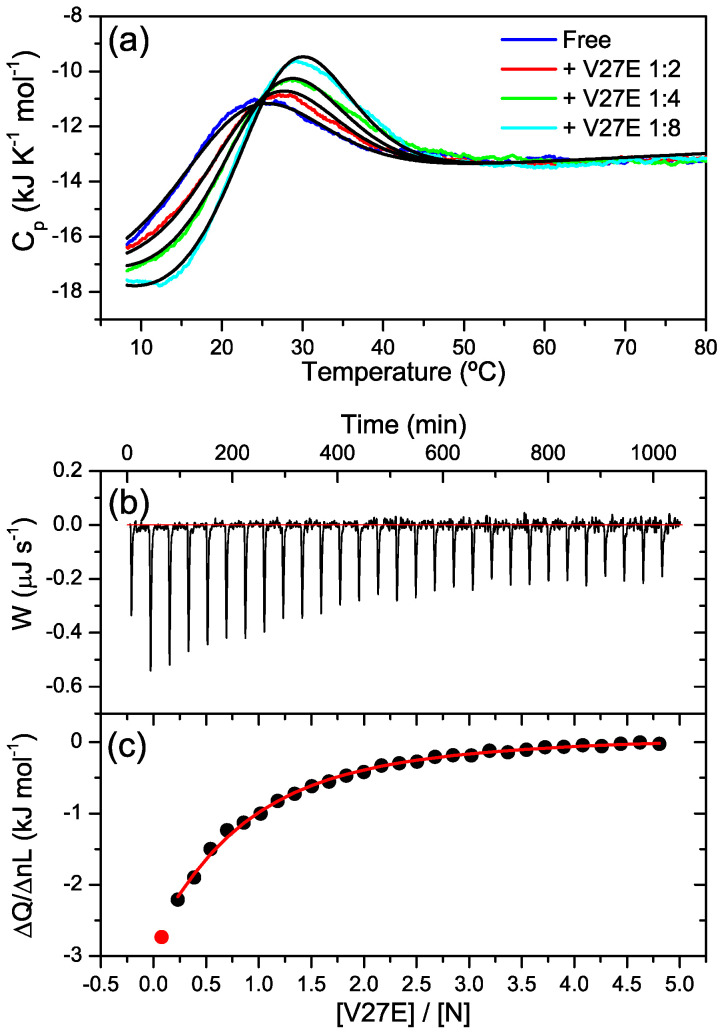
Binding of the HR2 V27E peptide to the CoVS-HR1-N protein. (**a**) DSC thermograms of CoVS-HR1-N at pH 7.4 in the presence of the V27E peptide at varying molar ratios. The protein concentration was 30 μM in all experiments. The black lines represent the global fitting of the curves using a model of two-state unfolding coupled with binding. (**b**) ITC thermogram of 30 μM of CoVS-HR1-N titrated with V27E peptide at 25 °C and pH 7.4. (**c**) Binding isotherm calculated from the thermogram of (**b**). The symbols represent the binding heats normalized per mole of added peptide. The red line represents the fitting using a model of n independent and identical binding sites.

DSC experiments with CoVS-HR1-N at pH 7.4 in the presence of V27E at various protein:peptide molar ratios also showed clear temperature shifts and increases in the area of the unfolding peak, which can be attributed to protein–peptide binding (Figure 9a). The data were globally fit reasonably well using a model of 1:1 ligand binding coupled with two-state unfolding (NL ⇔ N + L ⇔ U + L) [39]; however, the data could not be adequately fit with a model of binding to protein dimers. The apparent dissociation constant derived from the DSC analysis was about 160 μM at 25 °C. The binding of V27E to the N miniprotein was confirmed by ITC experiments at 25 °C (Figure 9b). The binding isotherm was not sigmoidal and could not be fit using a 1:1 stoichiometry. However, the data were well-fit with a binding stoichiometry fixed at 0.5 (Table 1), probably because at 25 °C, part of the N miniprotein is unfolded and therefore unable to bind the peptide. Thus, the binding parameters obtained from the DSC and ITC fittings do not need to agree, as they represent different processes. Furthermore, the simple models used to fit the data did not account for the influence of self-association of the protein upon the binding process. Nevertheless, these results show that the isolated N subdomain can bind the complementary HR2 peptide, albeit with considerably reduced affinity compared to the complete L1 and L2 proteins.

We also analyzed the binding the V27E peptide to the full-length L1 and L2 proteins by ITC (Figure S7a,b). This shorter peptide bound to L1 with similar binding enthalpy but lower affinity than the V39E peptide, although the binding stoichiometry arising from the fitting was considerably lower (Table 1). Binding of the same V27E peptide to L2 gave rise to minimal heat, and the binding isotherm did not allow for a determination of the thermodynamic parameters of binding, indicating very weak binding. These results indicate that the full HR2 region is needed to establish a high-affinity HR1–HR2 interaction.

In contrast to the N subdomain, the presence of the V19E peptide did not alter the thermal unfolding profile of CoVS-HR1-C as measured by DSC (Figure S8a), and an ITC titration of the C miniprotein with the V19E peptide did not produce measurable heats (Figure S8b,c), confirming that the C subdomain could not interact in isolation with its complementary HR2 region.

These results clearly indicate that the unstable N-terminal domain of HR1 contains the main HR2-binding determinants, whereas the highly stable C-terminal half has an intrinsically low affinity for its complementary HR2 region. However, the binding affinity between the full-length HR1 and HR2 regions was strongly increased, suggesting a cooperativity mechanism between the interactions in the two subdomains.

### 2.7. In Vitro SARS-CoV-2 Inhibition

**Figure 10:**
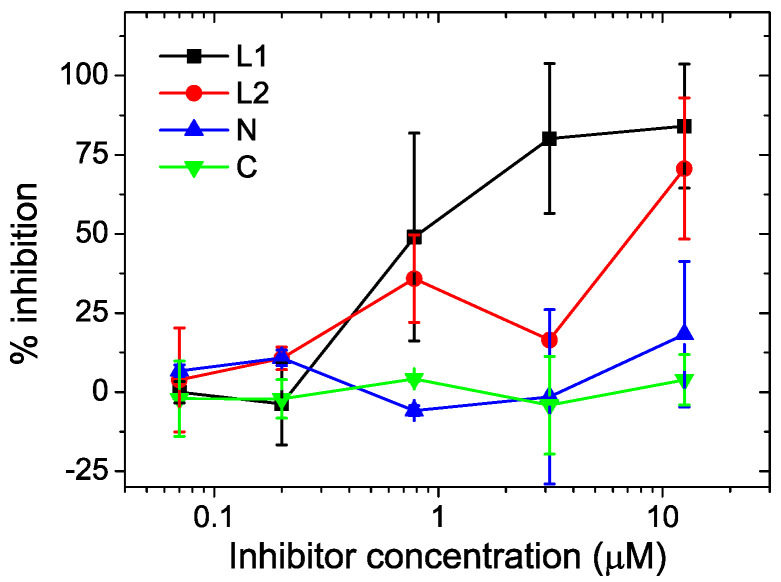
In vitro inhibition of SARS-CoV-2 infection of Vero 76 cells by CoVS-HR1 proteins. Vero 76 cells were infected by primary viruses (B1 D614G genotype) in the presence of CoVS-HR1 proteins at varying concentrations. The percentage of inhibition was calculated according to the reduction in percentage of infected cells treated with inhibitor compared to the percentage of infected untreated control cells. The percentage of infected cells in each well was recorded by measuring the number of infected cells (labeled by antinucleocapside antibody) and the total number of cells (by Sytox Green staining) using an imaging cytometer. The data and error bars correspond to the mean and standard deviation from two independent experiments.

We tested the capacity of the four proteins to inhibit SARS-CoV-2 cell infection of Vero 76 cells in vitro. Primary wild-type (WT) B1 (D614G) strain isolated from a SARS-CoV-2-infected individual was used for infection. The L1 and L2 proteins showed inhibitory activity in the two independent experiments, with IC50 values of 0.9 µM and 9 µM, respectively (Figure 10). On the contrary, the N and the C miniproteins did not show detectable activity, which is consistent with the stronger HR2 binding affinity of the L1 and L2 proteins compared to the weak or undetectable affinities of the shortened miniproteins. Although the L2 protein showed higher apparent affinity for the HR2 peptide than L1, the latter demonstrated a stronger inhibitory activity. None of the proteins exhibited significant toxicity to the cells, as shown by the total number of cells measured in the treated wells by Sytox Green staining. These results constitute a proof of concept of the feasibility of targeting the HR2 region with chimeric mimics of HR1.

## 3. Discussion

Protein or peptide mimics of the gp41 HR1 region have been shown to constitute potent inhibitors of HIV-1, owing to their capacity to tightly bind the HR2 region and interfere with virus–cell fusion [24,25,26,31]. Our results, together with other reports in the literature [22,23], clearly indicate that HR1 mimetic constructs can also inhibit SARS-CoV-2 by binding to HR2 and presumably blocking the membrane fusion process at some of its stages. According to the classical spring-loaded model of spike-mediated fusion (Figure 1a), HR1 forms an exposed trimeric coiled-coil bundle in the extended fusion intermediate of S2, which also transiently exposes HR2 to the action of inhibitors. This unstable intermediate then evolves to the 6-helix bundle structure that promotes fusion. In the framework of this model, constructs mimicking a stable HR1 trimeric bundle would be able to compete with the intramolecular HR1–HR2 interaction and block fusion. In support of this inhibition mechanism, vaccination with stabilized gp41 HR1 trimers can elicit neutralizing antibodies against HIV-1 [40] and HIV-1 in infected patients to elicit neutralizing antibodies that target exposed epitopes of trimeric HR1 [41]. Moreover, a strong correlation between conformational stability and HIV-1 inhibitory activity has been observed in gp41 HR1-mimetic proteins [31,32,33]. Other researchers have suggested an alternative model, in which both HR1 and HR2 regions transiently interact with the membranes, destabilizing them and facilitating fusion [42]. In this model, six-helix bundle formation only occurs in a late stage or after initiation of pore formation. Irrespective of the mechanistic details, the highly conserved HR2 region is crucial for membrane fusion; therefore, constructs that bind and sequester it, such as our CoVS-HR1 constructs, are capable of blocking SARS-CoV-2 infection.

Among the available approaches to stabilize HR1-based polypeptides in a stable coiled-coil trimeric structure, we previously reported that organizing three HR1 helices in an antiparallel orientation connected with short loops is very effective to create highly accurate structural mimics of an exposed HR1 region and that these mimics can potently and broadly inhibit HIV-1 [24,30,33]. Moreover, these mimetic proteins constitute very useful models to investigate the structural and thermodynamic determinants of the HR1–HR2 interaction [32,43,44].

Here, we applied a similar strategy to SARS-CoV-2. However, the molecular characteristics of the S2 HR1 region, with a considerably longer coiled coil than that of HIV-1 gp41, made it challenging to design protein mimics. Despite extensive amino acid engineering efforts to stabilize their antiparallel fold, the chimeric proteins did not behave as fully monomeric and cooperatively folded molecules, although they contain subdomains of varying stability and have a tendency to self-associate, forming trimers. This propensity for self-association could related to the considerable exposure of a hydrophobic surface area in the HR1 groove, which may mediate intermolecular interactions, although there is also a possibility that part of the HR1 helices, probably in the C-terminal region, could find an alternative more stable fold by trimerization. Moreover, the most N-terminal half of the coiled-coil is very unstable and shows molten globule-like characteristics [45,46], including a propensity for self-association typical of partially folded species [47,48]. These characteristics are reminiscent of the initial designs of our previous gp41 HR1 mimics, which also required considerable engineering to stabilize in a monomeric form [24]. We also observed that the N-terminal half of SARS-CoV-2 HR1 mimics is much less stable than the C-terminal subdomain, as observed in our previously reported gp41 HR1 mimics [31], with a highly unstable N-terminal subdomain that required more engineering to stabilize an antiparallel coiled-coil structure than the C-terminal subdomain.

Here, we demonstrated that the less stable N subdomain harbors the main determinants of HR2 binding, including the binding interface of the core HR2 helical region. In contrast, the C-domain does not show in isolation, binding to its complementary extended HR2 region. In the L1 and L2 proteins, the complete groove shows considerably higher binding affinity for the full-length HR2 V39E peptide. Furthermore, the shorter V27E peptide containing the HR2-binding core but devoid of the first ten HR2 residues that interact with the C domain shows a much weaker but significant binding for the L1 protein, indicating that the binding interface in the C domain is insufficient to mediate significant binding in isolation but that it cooperates significantly with the N subdomain, contributing to the overall HR1–HR2 binding energy. This is a clear indication of the existence of a cooperative distribution of binding energy across regions of the HR1–HR2 interface. Similar observations have been reported for the HIV-1 gp41 HR1–HR2 interaction [44,49].

Despite the low stability and molten globule characteristics of the CoVS-HR1-N miniprotein, it is still capable of binding HR2, albeit with low affinity. However, this was not reflected by a detectable capacity to inhibit the virus in our experiments. A potential advantage of such a miniprotein is its small size, which reduces possible steric impairment to access selected and partially accessible HR2 regions. In the HIV-1 spike, the first half of HR2 is buried and engaged in interactions stabilizing the complex with gp120, whereas the second half is more accessible [50], which might limit the activity of protein-based HR1 inhibitors that can only act on the transient prefusion intermediate once the spike has been activated and the whole HR2 region becomes exposed [51,52]. In coronaviruses, the highly conserved HR2 region forms part to the spike stalk, which appears to be shielded by the dynamics of flexible glycans that limit accessibility to inhibitors and antibodies [53]. Small HR1 mimics could therefore have an advantage over larger constructs because of less restricted access to their target. We recently reported that stabilizing gp41 HR1 small miniproteins by disulfide bonds resulted not only in a considerable increase in binding affinity for HR2 but also in strong improvement in HIV-1 inhibitory activity [33]. This strategy, in addition to other engineering approaches, can be used to produce stable N-HR1 miniproteins with improved stability and inhibitory activities.

Despite their conformational and self-association issues, the full-length L1 and L2 proteins show significant and reproducible inhibitory activity against in vitro infection of cells by real SARS-CoV-2 virus by targeting HR2. Inhibition of coronavirus infection has also been reported using stabilized trimers of HR1 derived from the HIV-1 gp41 sequence [21]. Furthermore, a recent report described a five-helix construct consisting of three HR1 and two HR2 fragments that leave an exposed HR1 groove [22], binds an HR2 peptide with high affinity and can inhibit S-mediated cell–cell fusion and cell infection by various pseudotyped SARS-CoV-2 variants, as well as WT and Delta real SARS-CoV-2 viruses. In a second recent paper, trimers of S2 HR1 polypeptides stabilized by conjugation to a foldon sequence (HR1MFd) [23] were reported to show inhibitory activity against SARS-CoV-2, SARS-CoV-2 variants of concern (VOCs), SARS-CoV and MERS-CoV. Although the cells and the assays used to quantify infection in these studies differ from those used in the present study, the inhibitory activity we observed for L1 and L2 constructs is slightly lower than that of the five-helix construct (IC50s ≈ 300 nM) but similar to the HR1MFd (IC50s ≈ 1–3 μM).

The inhibitory activity of CoVS-HR1-L1 and -L2 proteins may be compromised by their tendency to self-associate, which might result in a partial occlusion of the HR1 groove and/or in a size increase that may impair accessibility to HR2 in the virion-cell context. In addition, the inhibitory activity of L2 is lower than that of L1, although the former has an apparently higher affinity for the full V39E HR2 peptide. In contrast, L2 shows undetectable binding for the shorter V27E peptide that harbors the core HR2 binding region, whereas L1 maintains a significant binding affinity for this peptide (Table 1, Figure S7), which may be related to the slow binding observed for the L2 variant, which appears to be in a metastable state an unable to bind HR2 as a result of oligomerization. Our results indicate that HR2 binding drives L2 to a monomeric state (Figure 5e,f). It is therefore possible that the necessary oligomer dissociation and an accompanying structural reorganization of the protein could set an energy barrier to form the complex. This could kinetically limit its capacity to bind a transiently exposed HR2 region in the viral Spike HR2. Moreover, the N-terminal domain, which contains the core HR2-binding determinants in HR1, shows low stability and molten globule-like characteristics, further compromising HR2 binding. Nevertheless, the inhibitory activity observed against real viruses is comparable to that of other constructs, especially for L1, supporting the feasibility of targeting the HR2 region of S2 as an effective way to inhibit SARS-CoV-2 infection. Further designs of newly improved CoVS-HR1 molecules are being developed to reduce self-association and improve conformational stability, which should result in improved inhibitory capabilities.

The targeting of HR1 by HR2 peptides has also been reported as a potent and broad approach to inhibit SARS-CoV-2 fusion [15,54,55]. However, HR1 is less conserved than HR2 between various SARS-CoV-2 variants [22] and other coronaviruses [18]. Moreover, as highly stable folded proteins, HR1 mimetics should have longer half-life than HR2 peptides, which are rapidly degraded by host proteases. As single-polypeptide chains, a major advantage of our HR1 mimetics is their relatively small molecular size and easy production by overexpression in recombinant form, which can be scaled at a lower cost than synthetic peptides and without the need for chemical modification or addition of external elements to stabilize the desired structure. Moreover, these chimeric proteins are highly soluble and highly stable, facilitating their formulation as potential therapeutics.

## 4. Materials and Methods

### 4.1. Computational Modelling

Modeling was carried out using SwissPDBviewer [56] and YASARA structure [34]. As template, we used the published X-ray crystal structure of the six-helical bundle formed by HR1 and HR2 in the S2 post-fusion structure (PDB id. 6LXT [15]).

### 4.2. Proteins and Peptides

The DNAs encoding the protein sequences were synthesized and cloned into pET303 expression vectors by Thermo Fisher Scientific (Waltham, MA, USA). The sequences included an N-terminal methionine and a C-terminal 6×histidine tag with the sequence GGGGSHHHHHH. *E. coli* bacteria (BL21(DE3)) were transformed with the plasmids and cultured at 37 °C in the presence of 30 μg·mL^−1^ Ampicillin (Sigma-Aldrich, MO, USA). Protein expression was induced with 0.5 mM IPTG, and the cells were cultured overnight at 27 °C. Cells were collected by centrifugation and resuspended in lysis buffer (50 mM Tris/HCl, 500 mM NaCl, 1 mM EDTA, 1 mM β-mercaptoethanol) containing a cocktail of protease inhibitors (Sigma-Aldrich, St. Louis, MO, USA). The cells were then lysed with three 2 min ultrasonication cycles on ice, and the soluble and insoluble fractions were separated by 30 min ultracentrifugation at 4 °C at 30,000 rpm. The proteins were purified from the supernatant fraction by NTA-affinity chromatography. A second polishing step was carried out by ion exchange chromatography on a HiTrap SP Sepharose XL column (Amersham GE Healthcare, UK). Protein purity was assessed by SDS-PAGE, and the identity of each protein was confirmed by mass spectrometry (Figures S9 and S10). Pure proteins were dialyzed against slightly acidified water and stored frozen at −80 °C.

Synthetic peptides derived from the S2 HR2 sequence were acquired from Genecust (Luxembourg), with a purity >95%. All the peptides were C-terminally tagged with an SGGY sequence to confer UV absorption at 280 nm and were N-acetylated and C-amidated.

For biophysical characterization, the protein solutions were extensively dialyzed against the appropriate buffer and centrifuged at 4 °C for 30 min in a bench microfuge before concentration measurement. For studies at varying pH values, appropriate buffers were employed at a 50 mM concentration (glycine/HCl for pH 2.5; sodium acetate for pH 4 and pH 5; sodium cacodylate for pH 6; sodium phosphate for pH 7.4; and sodium bicarbonate for pH 9.4). Stock peptide solutions were freshly prepared by weighting the necessary amount of lyophilized peptide and dissolving it in the appropriate buffer. Thereafter, the pH was checked and corrected, if necessary, with HCl or NaOH solutions, and the final peptide concentration was measured spectrophotometrically. Protein–peptide mixtures were prepared by adding the appropriate volume of peptide stock solution to the previously prepared protein sample. Protein and peptide concentrations were measured by UV absorption at 280 nm, with extinction coefficients calculated according to their respective amino acid sequences using the ExPasy ProtParam server (https://web.expasy.org/protparam/, accessed on 5 June 2022) [57]. All samples were freshly prepared and used immediately in experiments.

### 4.3. Circular Dichroism (CD)

CD measurements were carried out with a Jasco J-715 spectropolarimeter (Tokyo, Japan) equipped with a temperature-controlled cell holder. Measurements of the far-UV CD spectra (260–200 nm) were conducted with a 1 mm path length quartz cuvette. Spectra were recorded at a scan rate of 100 nm/min, 1 nm step resolution, 1 s response and 1 nm bandwidth. The resulting spectrum was usually the average of 5 scans. Each spectrum was corrected by baseline subtraction using the blank spectrum obtained with the buffer, and the CD signal was normalized to molar ellipticity ([θ], in deg·dmol^−1^·cm^2^). Thermal scans were performed by measuring the CD signal at 222 nm as a function of temperature using a scan rate of 2 °C min^−1^. The protein concentration was typically 15 μM in these measurements.

### 4.4. Light Scattering

Dynamic light scattering (DLS) was measured with a DynaPro MS-X DLS instrument (Wyatt, Santa Barbara, CA, USA). Dynamics v6 software (Wyatt Technology Corporation, Santa Barbara, CA, USA) was used for data collection and processing. Sets of DLS data were measured at 15 μM and 25 °C unless stated specifically, with an average of 50 acquisitions and an acquisition time of 10 s.

Static scattering intensities were measured with a DynaPro MS-X DLS instrument (Wyatt, Santa Barbara, CA) or a Malvern μV instrument (Malvern Panalytical, Malvern, UK) at 25 °C in 50 mM sodium phosphate buffer (pH 7.4) at varying protein concentrations in the range of 0.2–4.5 g L^−1^. The intensities were analyzed using a Debye plot as represented by Equation (1)

(1) $$K\cdot c/R_{90}=1/M_{w}+2A_{2}c$$

which is valid for particles significantly smaller than the wavelength of the incident radiation, where *K* is the optical constant of the instrument, *c* is the particle mass concentration, *R*_90_ is the Rayleigh ratio of scattered to incident light intensity, *M_w_* is the weight-averaged molar mass, *A*_2_ is the 2nd virial coefficient that is representative of interparticle interaction strength and *M_w_* can be determined according to the intercept of the plot.

### 4.5. Differential Scanning Calorimetry

DSC experiments were carried out in a MicroCal PEAQ-DSC microcalorimeter equipped with autosampler (Malvern Panalytical, Malvern, UK). Scans were normally run from 5 to 130 °C at a scan rate of 90 °C·h^−1^ and a protein concentration of 30 μM unless stated specifically. Instrumental baselines were recorded before each experiment with both cells filled with buffer and subtracted from the experimental thermograms of the protein samples. Consecutive reheating runs were carried out to determine the reversibility of the thermal denaturation. The excess heat capacity (ΔC_p_) relative to the buffer was calculated from the experimental DSC thermograms using Origin software (OriginLab, Northampton, MA, USA) and normalized per mole of protein.

### 4.6. Isothermal Titration Calorimetry

ITC measurements were carried out with a Microcal VP-ITC calorimeter (Malvern Panalytical, Malvern, UK). The proteins were titrated with 25 injections of 5 μL peptide solution at 480 s intervals. Protein concentration in the cell was generally approximately 10 μM, whereas the peptide concentration in the syringe was typically between 200 μM and 400 μM. The experiments were carried out in 50 mM phosphate buffer (pH 7.4) at 25 °C. The experimental thermograms were baseline-corrected, and the peaks were integrated to determine the heat produced by each ligand injection. Residual heat resulting from non-specific binding or ligand dilution was estimated according to the final peaks of the titrations. Each heat was normalized per mole of injected ligand. The resulting binding isotherms were fitted using a binding model of independent and equivalent sites, allowing for the determination of the binding constant (K_b_), the binding enthalpy (ΔH_b_) and the binding stoichiometry (n).

### 4.7. Virus Inhibition Assays

One day prior to infection, Vero 76 cells were plated on a 96-well plate at 12,500 cells/well. Then, 50 µL of serial 4-fold dilutions of covS-HR1 proteins (2-fold concentrated) and 50 µL WT SARS-CoV-2 viruses (B1 D614G genotype) at multiplicities of infection (MOI) of 80 were added to the cells and incubated for 2 days. Cells were fixed with methanol for 20 min, washed with PBS and stained with antinucleocapside antibody (Genetex GTX135357) at 1/200 dilution in permwash (B&D) for 45 min at room temperature. Nucleocapside-positive cells were revealed with a donkey anti-rabbit monoclonal Ab (Alexa 647; A31573, Invitrogen, Waltham, MA, USA) diluted at 1/200 in PBS 5% FCS for 45 min at room temperature. In parallel, total cells were detected by Sytox green (S7020, Invitrogen) staining. Total cells (Sytox green positive) and infected cells (nucleocapside-positive) were counted using a SpectraMax MiniMax imaging cytometer (Molecular Devices LLC). The percentage of infected cells in each well was calculated according the number of infected cells relative to the total number of cells. Thereafter, the percentage of inhibition was calculated by comparing the percentage of infected cells in treated wells relative to the percentage of infected cells in untreated control wells. The 50% inhibitory concentration (IC50) was estimated by fitting the data using Hill’s sigmoidal function.

## Figures and Tables

**Table 1:** Apparent thermodynamic parameters of binding of the HR2 peptides to CoVS-HR1 proteins measured by ITC at 25 °C. The parameters correspond to the best fittings of the binding isotherms using a binding model of n identical and independent sites.

Protein	Peptide	K_d_ (μM)	ΔH_b_ (kJ·mol^−1^)	n
CoVS-HR1-L1	V39E ^(a)^	1.03 ± 0.24 ^(c)^	−33.5 ± 2.3	0.74
CoVS-HR1-L2	V39E	0.13 ± 0.04	−43.6 ± 1.8	0.66
CoVS-HR1-L1	V27E ^(b)^	4.1 ± 0.7	−37 ± 8	0.25
CoVS-HR1-N	V27E	28 ± 3	−7.7 ± 0.6	0.5 ^(d)^

^(a)^ HR2-derived peptide V39E encompassing residues Val1164-Glu1202. ^(b)^ HR2-derived peptide V27E encompassing residues Val1176-Glu1202. ^(c)^ Uncertainties in the parameters correspond to 95% confidence intervals of the parameters. ^(d)^ Parameter fixed in the fittings.

## Data Availability

The data presented in this study are available upon request from the corresponding author.

## Supplementary Materials

The supporting information can be downloaded at: https://www.mdpi.com/article/10.3390/ijms232415511/s1.
