# Supplementary material for: Exploring Highly Conserved Regions of SARS-CoV-2 Spike S2 Subunit as Targets for Fusion Inhibition Using Chimeric Proteins
Source: Int J Mol Sci. 2022 Dec 7;23(24):15511. doi: 10.3390/ijms232415511 (PMC9778920; doi:10.3390/ijms232415511)
Supplement: Supplementary file 1 [file ijms-23-15511-s001.zip › ijms-1863250-supplementary.pdf]

*Supplementary Information to:*

# Exploring highly conserved regions of SARS-CoV-2 spike subunit S2 as targets for fusion inhibition using chimeric proteins

**Daniel Polo-Megías [1], Mario Cano-Muñoz [1], Alberto González-Berruezo [1], Géraldine Laumond [2], Christiane Moog [2,3] and Francisco Conejero-Lara [1,*]**

[1] Departamento de Química Física, Instituto de Biotecnología y Unidad de Excelencia de Química Aplicada a Biomedicina y Medioambiente (UEQ), Facultad de Ciencias, Universidad de Granada, 18071 Granada, Spain.

[2] Laboratoire d'ImmunoRhumatologie Moléculaire, Institut National de la Santé et de la Recherche Médicale (INSERM) UMR_S 1109, Institut thématique interdisciplinaire (ITI) de Médecine de Précision de Strasbourg, Transplantex NG, Faculté de Médecine, Fédération Hospitalo-Universitaire OMICARE, Fédération de Médecine Translationnelle de Strasbourg (FMTS), Université de Strasbourg, F-67000 Strasbourg, France.

[3] Vaccine Research Institute (VRI), 94000 Créteil, France.

*** Correspondence to Francisco Conejero-Lara; e-mail: conejero@ugr.es**

**Table S1. Amino acid sequences of the reference HR1, HR2 (Uniprot P0DTC2) and the CoVS-HR1 variants.**

| | **Amino acid sequences** [(b)] |
|---|---|
| **HR1** | NVLYENQKLI ANQFNSAIGK IQDSLSSTAS ALGKLQDVVN QNAQALNTLV<br>KQLSSNFGAI SSVLNDILSR LDKVE |
| **V39E** [(a)] | VDLGDISGIN ASVVNIQKEI DRLNEVAKNL NESLIDLQE |
| **V27E** | VVNIQKEI DRLNEVAKNL NESLIDLQE |
| **V19E** | VDLGDISGIN ASVVNIQKE |
| **CoVS-HR1-L1**[(c)] | **D**VLYENQKLI AN**E**FNSAIGK IQDSLSSTAS ALGKLQD**K**VN QNAQ**K**LNTLV<br>KQLSSNFGAI SS**K**LNDILSR LDK**IQSRI**KD LRSLIDNL**K**S **E**IAGFNSSLQ<br>KVLTNL**R**Q**E**N QNV**E**D**E**LKGL ASATSSL**E**DQ IKG**E**ASNFQN **R**ILKQNEYLV<br>N**RPSPQ**NVLY ENQKLIANQF NSA**E**GKIQDS LSST**K**SALGK LQDVVNQN**K**Q<br>ALNTL**E**KQLS SNF**R**AISSVL NDI**E**SRL**Q**KV **Q** |
| **CoVS-HR1- L2** | **D**VLYENQKLI AN**E**FNSAIGK IQDSLSSTAS ALGKLQD**K**VN QNAQ**K**LNTLV<br>KQLSSNFGAI SS**K**LNDILSR LDK**GQLNP**KD LRSLIDNL**K**S **E**IAGFNSSLQ<br>KVLTNL**R**Q**E**N QNV**E**D**E**LKGL ASATSSL**E**DQ IKG**E**ASNFQN **R**ILKQNEYLV<br>N**QILGP**NVLY ENQKLIANQF NSA**E**GKIQDS LSST**K**SALGK LQDVVNQN**K**Q<br>ALNTL**E**KQLS SNF**R**AISSVL NDI**E**SRL**Q**KV **Q** |
| **CoVS-HR1-N** | **D**VLYENQKLI AN**E**FNSAIGK IQDSLSSTAS ALGKLQD**K**VN Q**GQLNP**QNV**E**<br>D**E**LKGLASAT SSL**E**DQIKG**E** ASNFQN**R**ILK QNEYLVN**QIL** **GP**NVLYENQK<br>LIANQFNSA**E** GKIQDSLSST **K**SALGKLQDV VNQN |
| **CoVS-HR1-C** | SALGKLQD**K**V NQNAQ**K**LNTL VKQLSSNFGA ISS**K**LNDILS RLDK**GQLNP**K<br>DLRSLIDNL**K** S**E**IAGFNSSL QKVLTNL**R**Q**E** NQNV**E**D**E**LK**N** **QILGPN**KLQD<br>VVNQN**K**QALN TL**E**KQLSSNF **R**AISSVLNDI **E**SRL**Q**KV**QW** |

[a] The HR2 peptides contained a C-terminal SGGY tag and were N-acetylated and C-amydated.
[b] The substituted amino acids from the reference sequence are highlighted in bold and the amino acids corresponding to the loops have been underlined.
[c] The three protein variants contained a N-terminal Methionine and a C-terminal polyhistidine tag of sequence GGGGSHHHHHH.

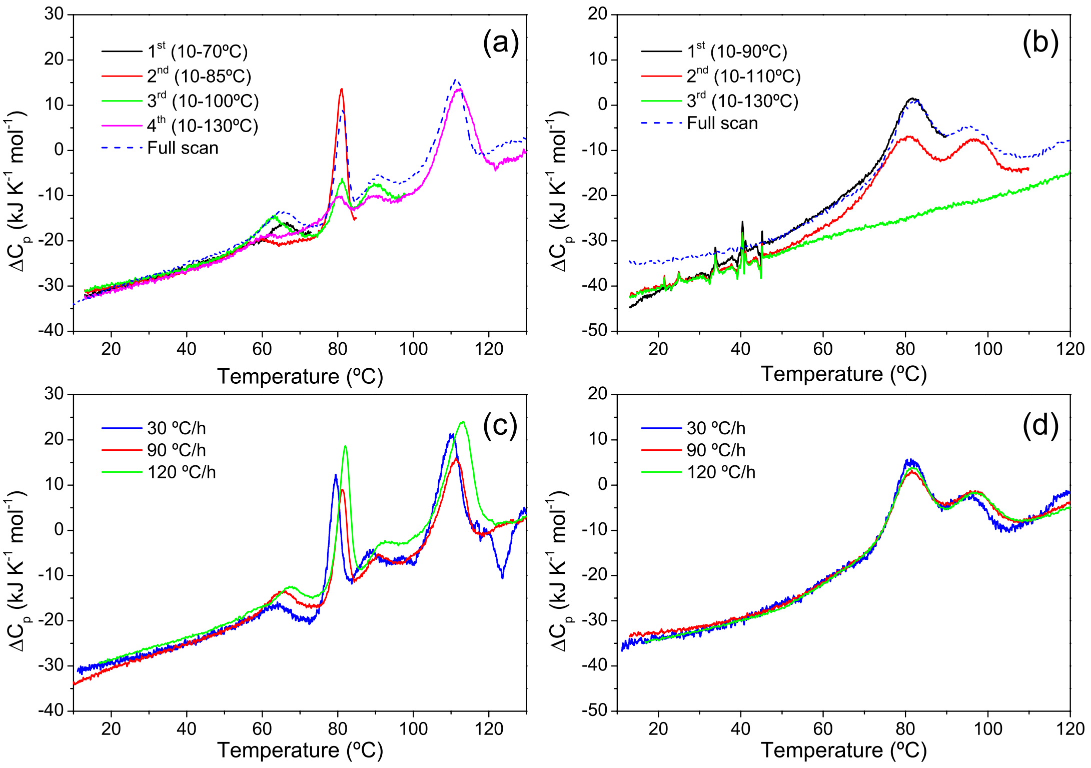


**Figure S1:** Reversibility of unfolding transitions of CoVS-HR1-L1 and L2 proteins. **(a)** and **(b):** Consecutive DSC scans carried out from 10°C up to the indicated temperatures with the same L1 (a) and L2 (b) samples at protein concentration of 30 μM and pH 7.4 at a scan rate of 90 °C/h. **(c)** and **(d):** Effect of the scan rate used in the DSC scans with L1 (c) and L2 (d) proteins under the same conditions used in the experiments of panels (a) and (b).

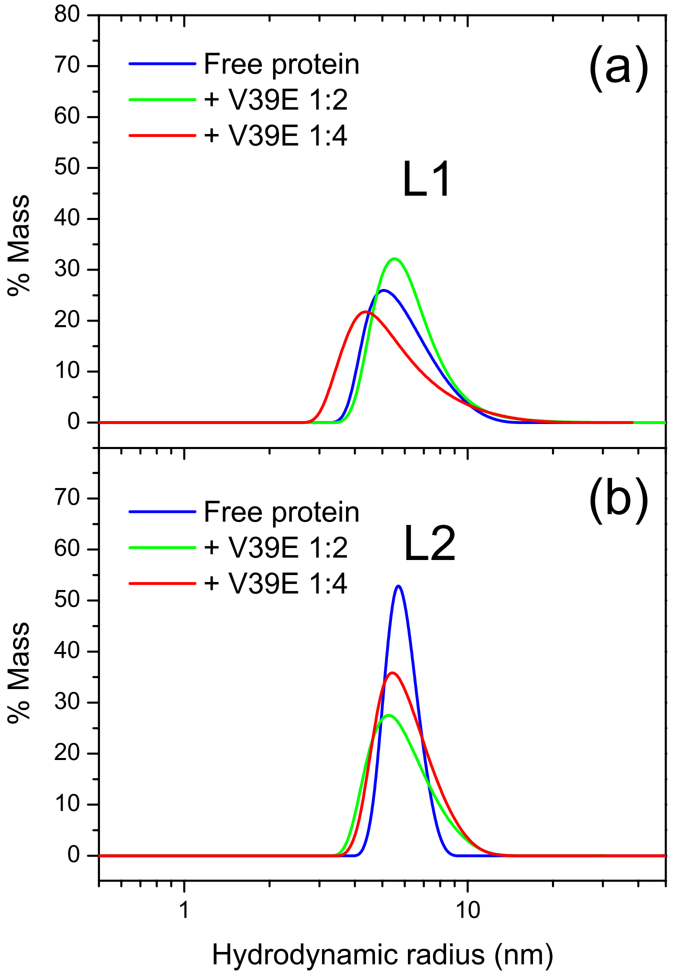


**Figure S2:** Effect of V39E peptide binding on the apparent hydrodynamic radii of CoVS-HR1 proteins measured by DLS at 25°C and pH 7.4. **(a)** L1 protein free at 30 µM and in presence of V39E at 1:2 and 1:4 molar ratio. **(b)** L2 protein free at 15 µM and in presence of V39E at 1:2 and 1:4 molar ratio.

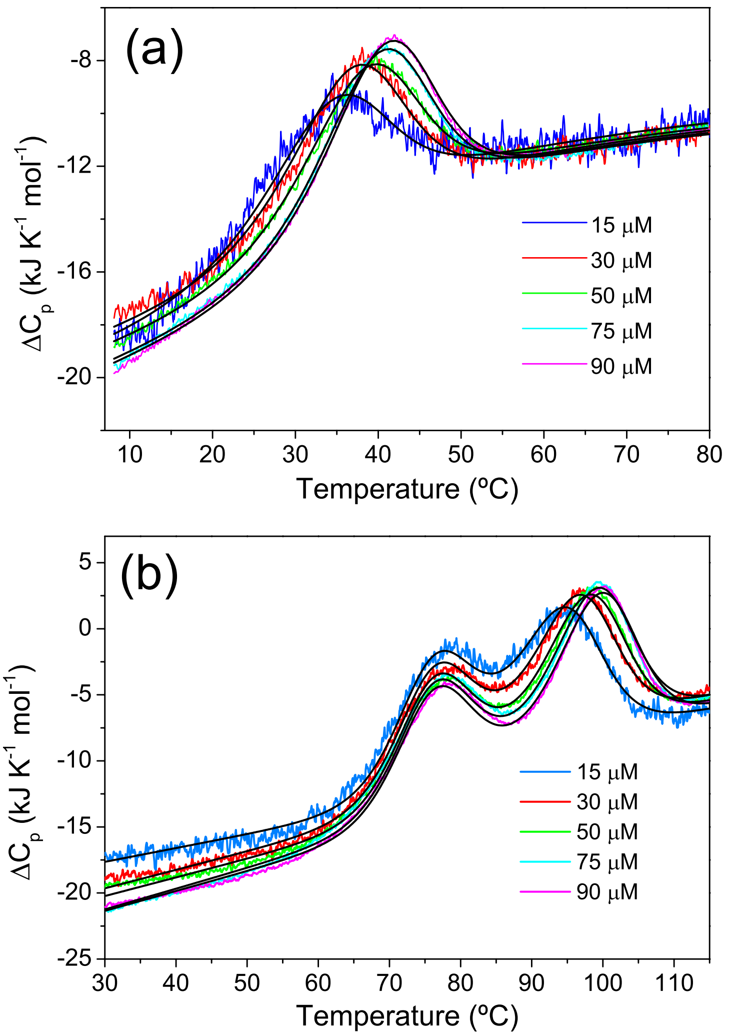


**Figure S3:** DSC scans of CoVS-HR1-N at pH 5 **(a)** and CoVS-HR1-C at pH 7.4 (b). The curves correspond to different protein concentrations, as indicated in the plot with different colors and have been normalized as heat capacity per mole of protein monomer. The experimental curves have been globally fitted using a $N_2 \Leftrightarrow 2U$ unfolding model for CoVS-HR1-N **(a)** and a $N_2 \Leftrightarrow I_2 \Leftrightarrow 2U$ unfolding model for CoVS-HR1-C **(b)** to account for the effect of protein concentration. The back lines correspond to the best fits.

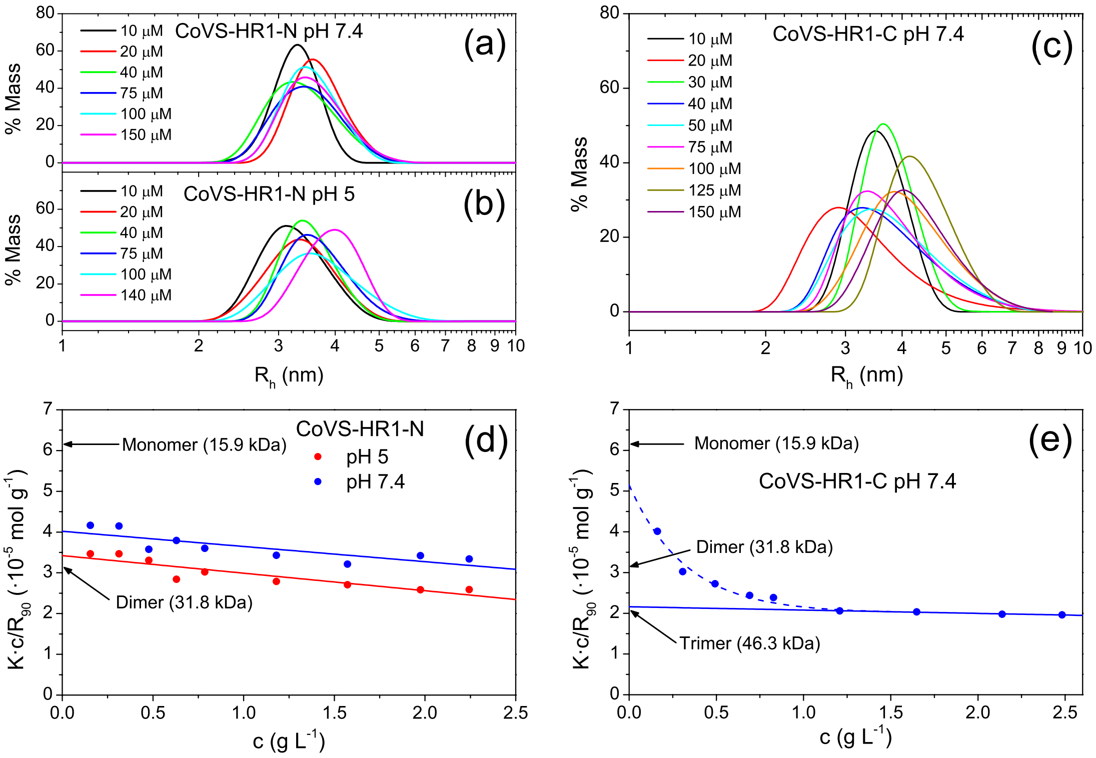


**Figure S4:** Molecular size characterization of CoVS-HR1 miniproteins. **(a) - (c):** Hydrodynamic radii distributions measured at 5°C and different protein concentrations by DLS for CoVS-HR1-N at pH 7.4 (a), CoVS-HR1-N at pH 5 (b) and CoVS-HR1-C at pH 7.4 (c). **(d) and (e)**: Debye plots corresponding to static light scattering measurements at different protein concentrations for CoVS-HR1-N at pH 7.4 and pH 5 (d) and CoVS-HR1-C at pH 7.4 (e). The intercepts indicate the inverse of the mass averaged molecular weight ($M_w$) of the particles.

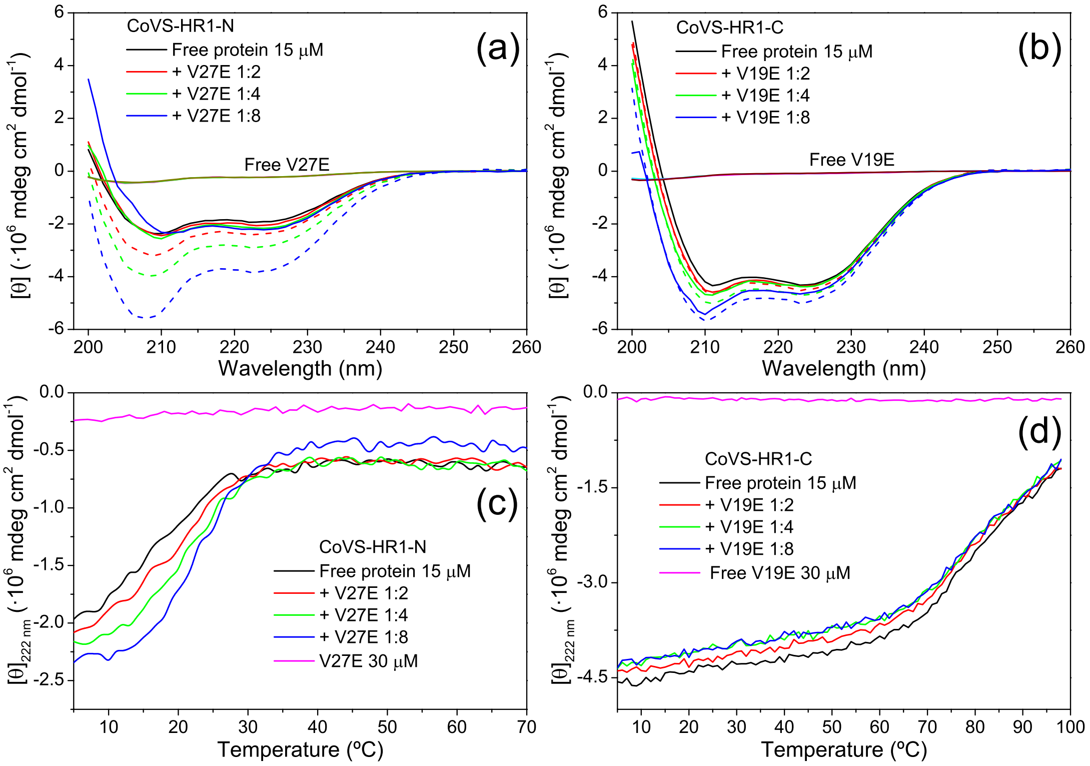


**Figure S5:** Influence of HR2 peptides on secondary structure and thermal unfolding of CoVS-HR1-N and CoVS-HR1-C miniproteins. **(a) and (b):** Far-UV CD spectra of N miniprotein (a) and the C miniprotein (b) in free form (black line) and in presence of the respective complementary HR2 peptides at different molar ratios, as indicated by the different colors. The spectra were measured at pH 7.4, 5°C and a 15 μM protein concentration and the peptide was added at 30 μM (red), 60 μM (green) and 120 μM (blue). The CD data have been normalized per mole of protein monomers. The theoretical spectra calculated as the sum of the free protein and peptide spectra at each protein:peptide ratio are represented by dashed lines with the respective colors. The CD spectra of the free peptides are also plotted as reference. **(c) and (d):** Thermal scans monitored by CD at 222 nm at a scan rate of 2 °C·h$^{-1}$ with each miniprotein in free form and in presence of each complementary HR2 peptide at different protein:peptide ratios. The experimental conditions used are the same as in panels (a) and (b).

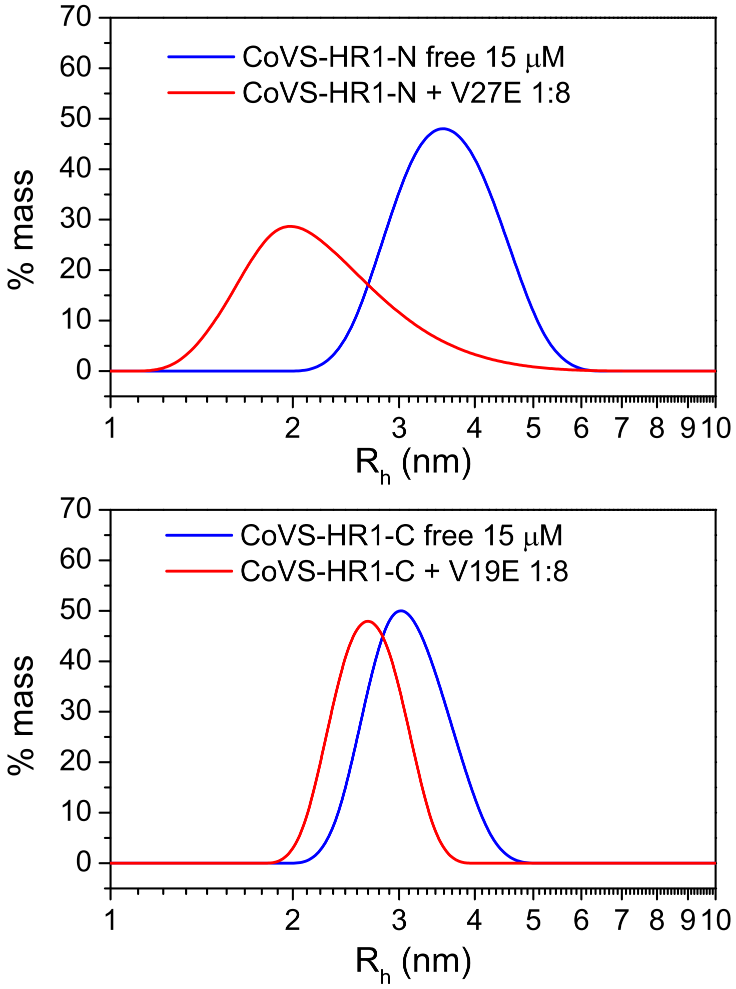


**Figure S6:** Effect of HR2 peptides binding on the apparent hydrodynamic radii of CoVS-HR1-N and C proteins measured by DLS at 5°C and pH 7.4. **(a)** CoVS-HR1-N protein free at 15 μM and in presence of V27E at 1:8 molar ratio. **(b)** CoVS-HR1-C protein free at 15 μM and in presence of V19E at 1:8 molar ratio.

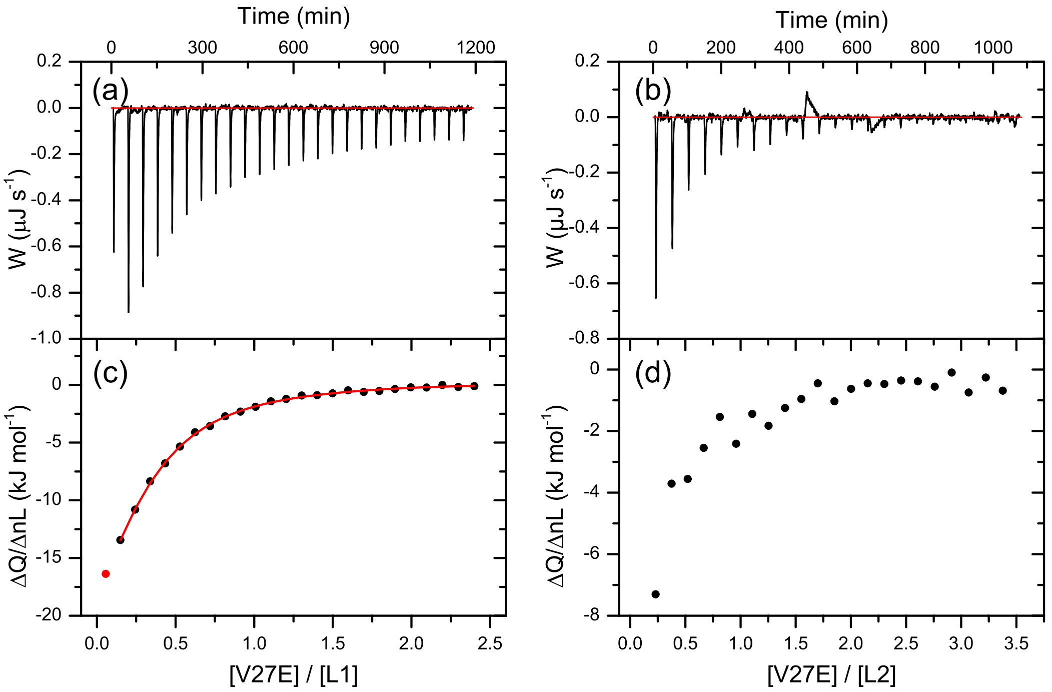


**Figure S7:** Binding of the HR2 V27E peptide to CoVS-HR1-L1 and CoVS-HR1-L2. **(a) and (b):** ITC thermograms of CoVS-HR1-L1 (a) and CoVS-HR1-L2 (b) titrated with V27E peptide at 25°C and pH 7.4. Protein concentrations in the calorimetric cell were around 10 μM. **(c) and (d):** Binding isotherms calculated from the thermograms of (a) and (b) respectively. The symbols represent the binding heats normalized per mole of added peptide in each injection. The red line in panel (c) represents the fitting using a model of n independent and identical binding sites.

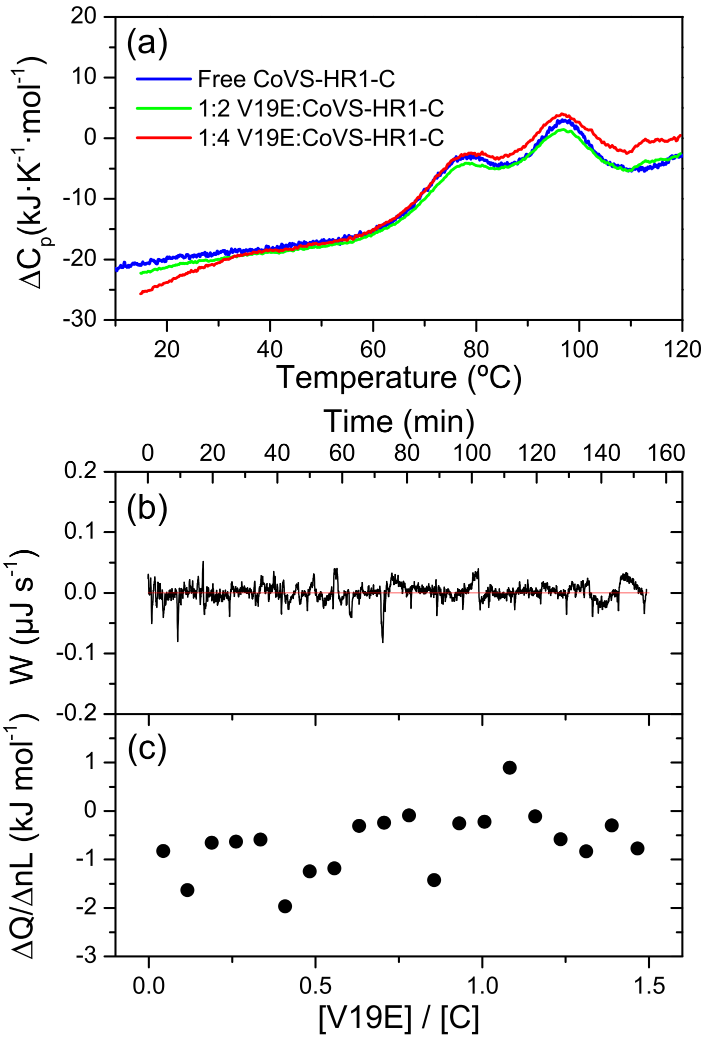


**Figure S8:** Binding of V19E to CoVS-HR1-C monitored by DSC and ITC. **(a)** DSC scans of CoVS-HR1-C at pH 7.4 in absence and in presence of the V19E peptide and different molar ratios. The protein concentration was 30 μM in all scans and the scan rate was 90 °C/h. The contribution of the heat capacity of the free peptide has been measured independently and subtracted from the scans to observe the net changes in the heat capacity of the protein. **(b)** Baseline corrected ITC thermogram corresponding to the titration of 10 μM CoVS-HR1-C with 200 μM V19E peptide in 5 μL injections. Temperature was 25°C and pH 7.4. **(c)** Normalized heats per mole of injected ligand calculated for each injection from the thermograms of (b).

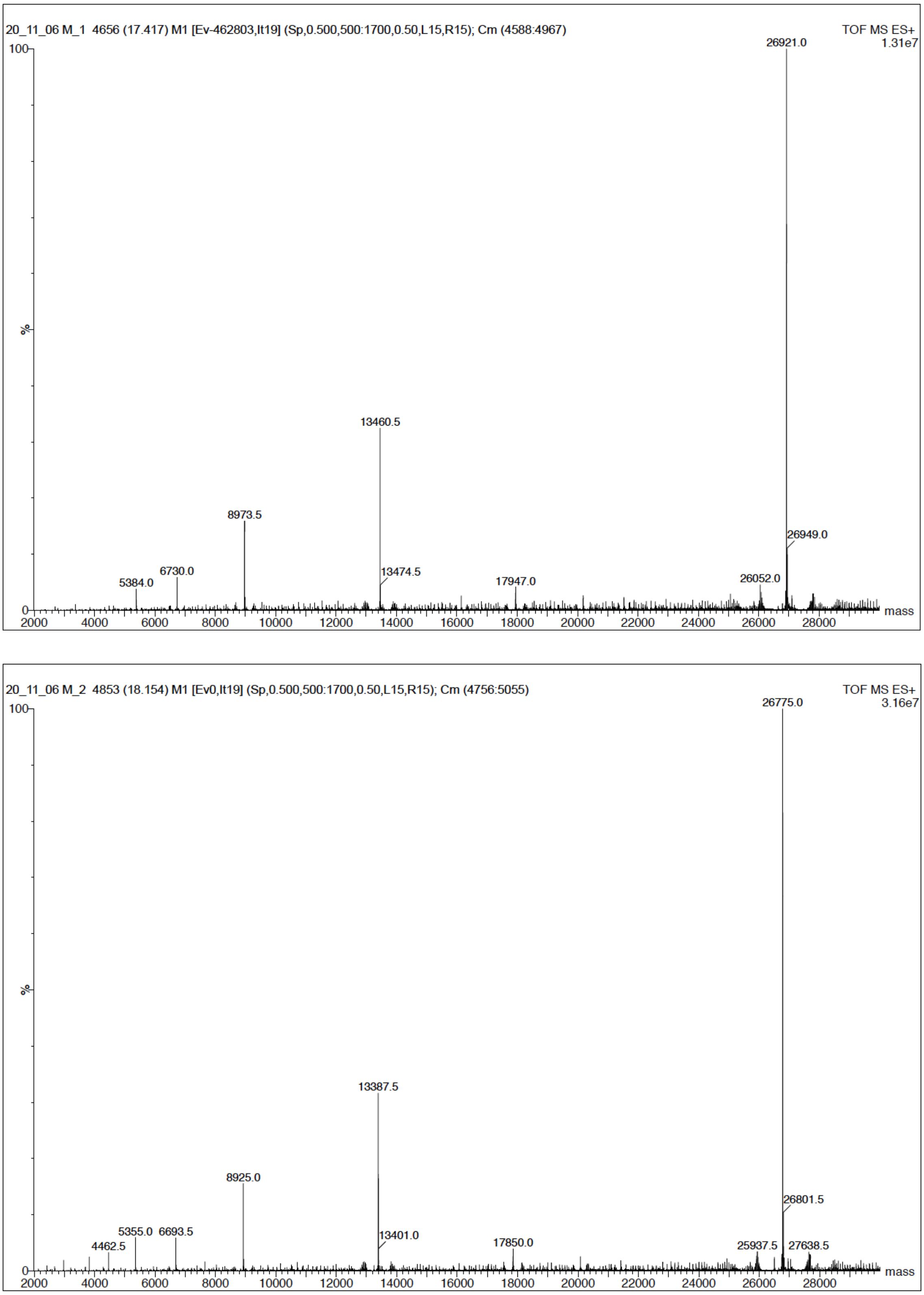


**Figure S9:** Mass spectrometry analysis of the CoVS-HR1 L1 and L2 proteins. Upper panel: CoVS-HR1-L1. Lower panel: CoVS-HR1-L2

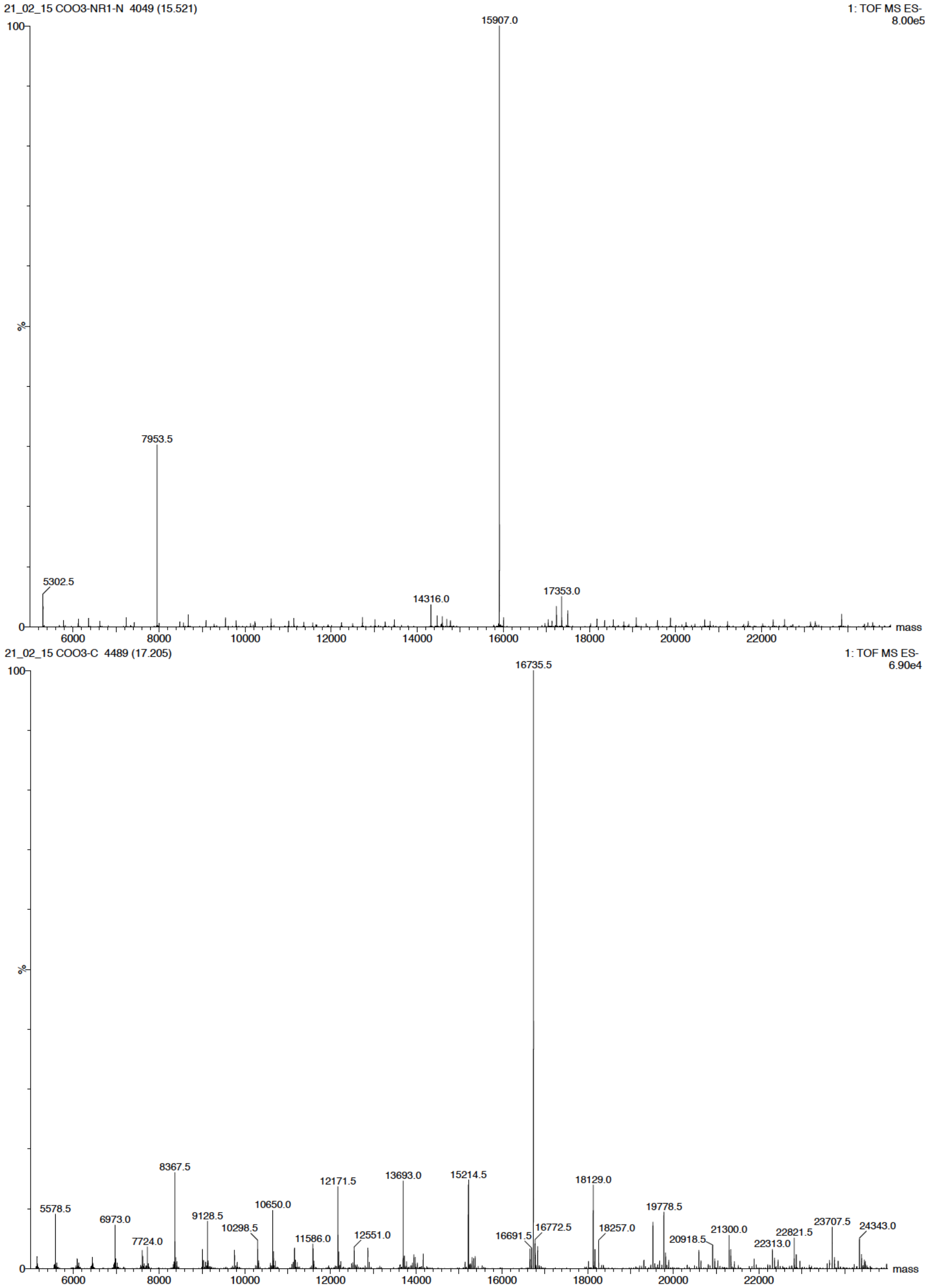


**Figure S10:** Mass spectrometry analysis of the CoVS-HR1 N and C proteins. Upper panel: CoVS-HR1-N. Lower panel: CoVS-HR1-C.
